# Supplementary figures and images for: Evidence of MHC class I and II influencing viral and helminth infection via the microbiome in a non-human primate
Source: PLoS Pathog. 2021 Nov 8;17(11):e1009675. doi: 10.1371/journal.ppat.1009675 (PMC8601626; doi:10.1371/journal.ppat.1009675)

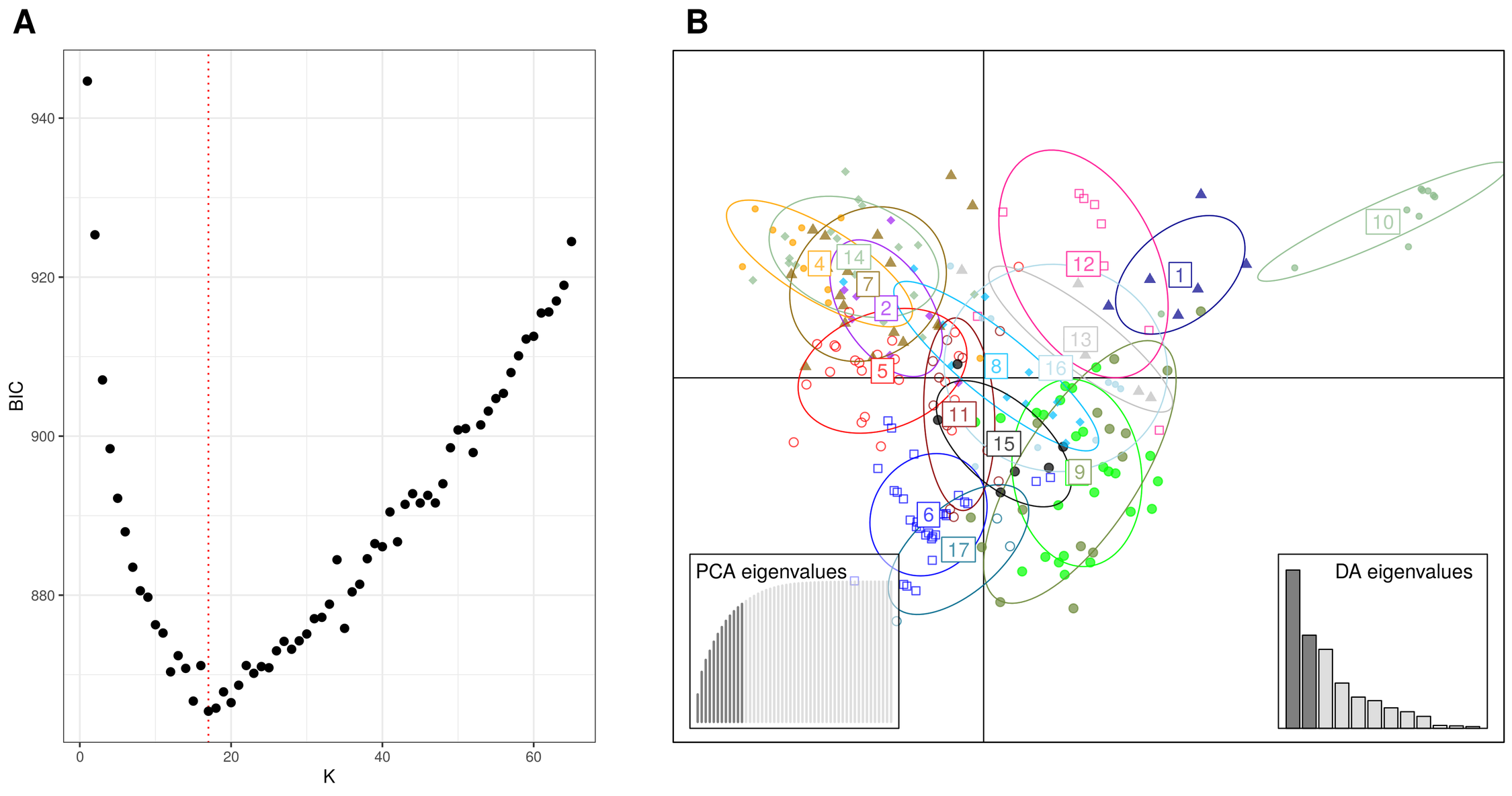

Supplement: S1 Fig — Clustering of MHC class I alleles into supertypes using discriminant analysis of principle components (DAPC) based on a matrix of physiochemical properties of the sites under positive selection (PSS) of each allele. (A) Number of clusters chosen using the find.clusters() function of the adegenet package [125]. The red dashed line shows (k) = 17. (B) Scatterplot of the first and second discriminant functions showing the supertype clusters. (TIF) [file ppat.1009675.s001.tif]

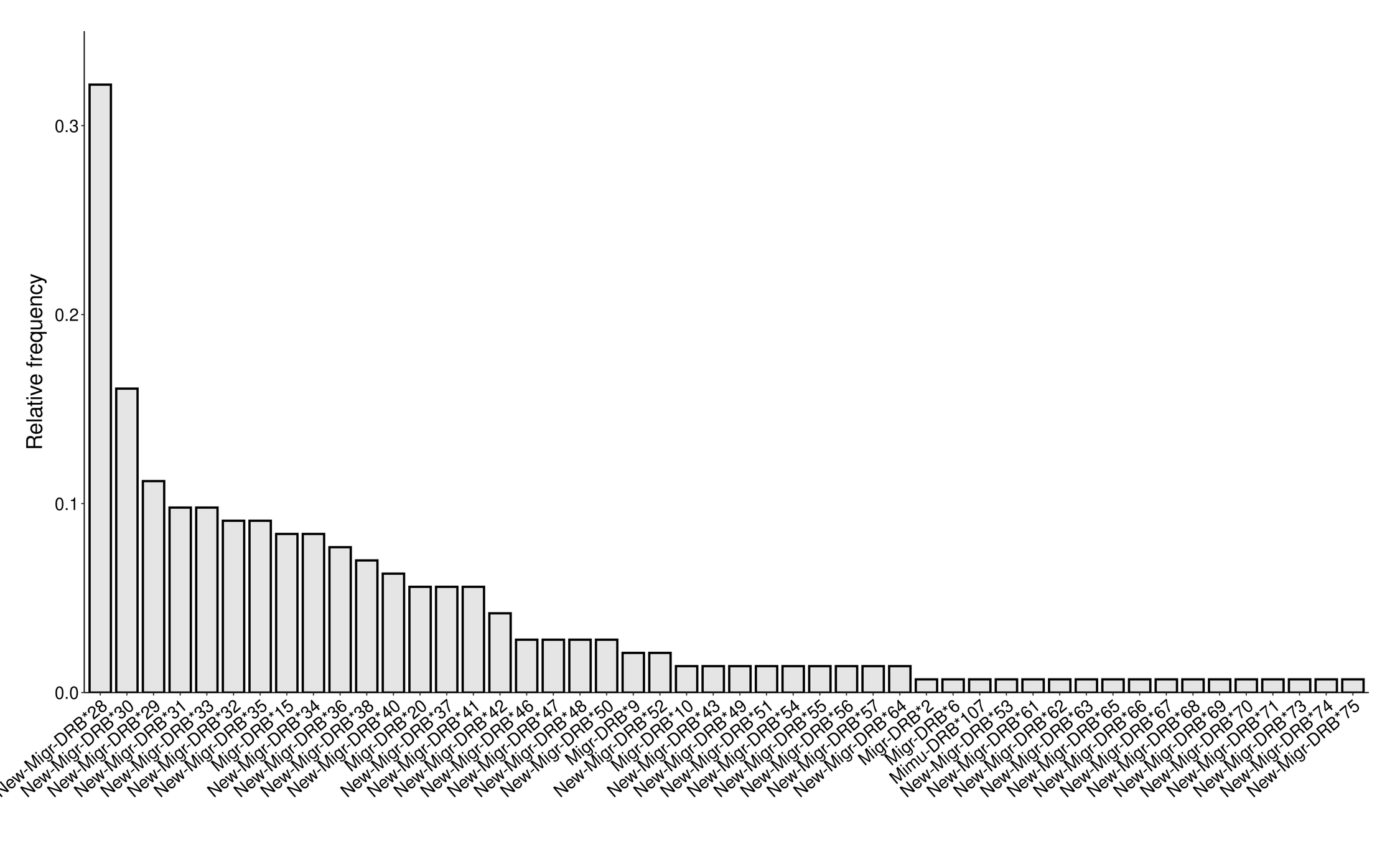

Supplement: S2 Fig — Relative frequencies of MHC class II DRB alleles in Microcebus griseorufus (n = 143). The identity of allele variants sharing identical amino−acid sequences at positive selected sites (PSS) is shown in parenthesis. Alleles Migr-DRB*28 to Migr-DRB*50(*58) were present in more than 3 individuals and the remaining rare alleles were not included in subsequent analyses. (TIF) [file ppat.1009675.s002.tif]

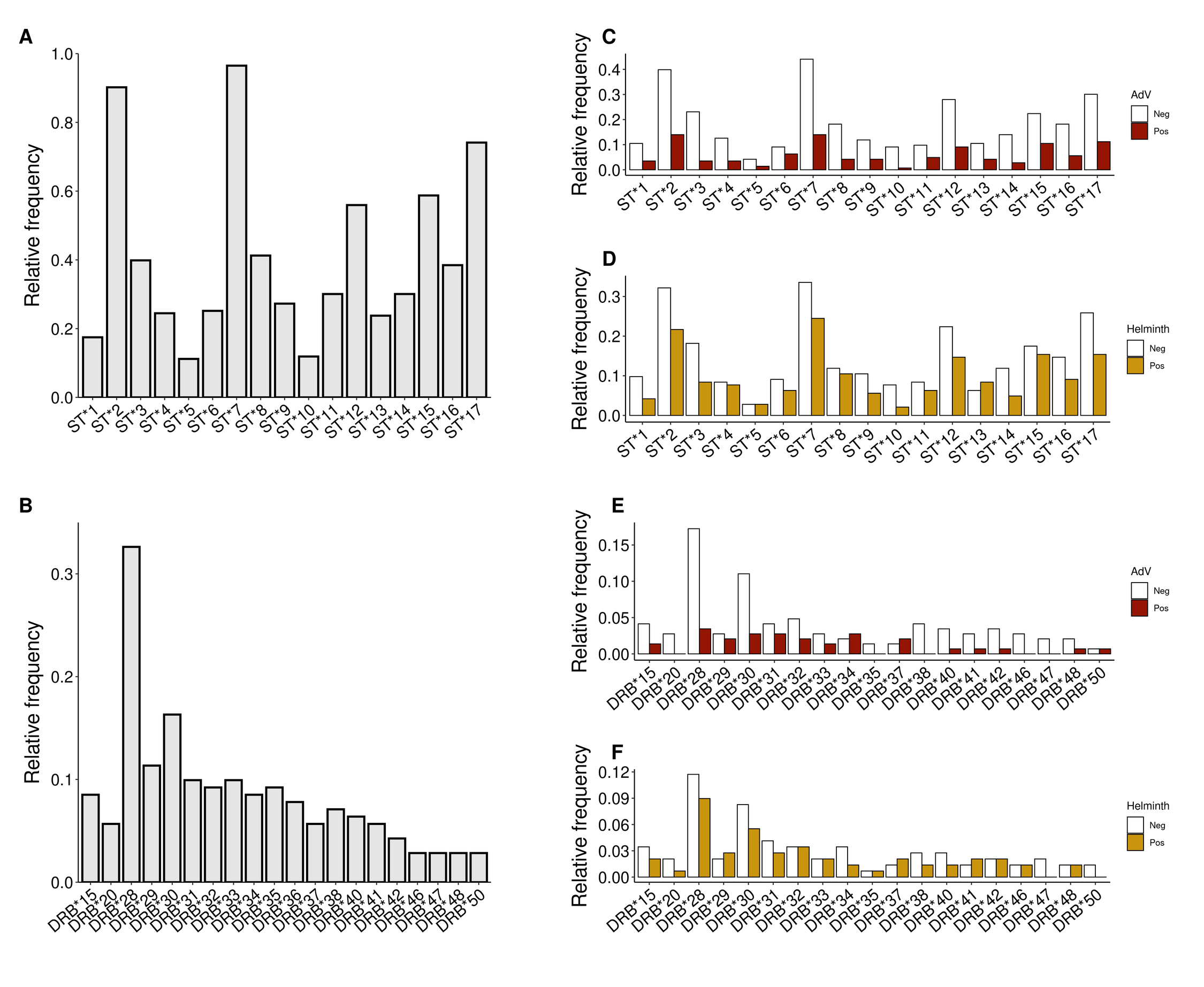

Supplement: S3 Fig — Relative motif frequencies of MHCI supertypes (A) and MHCII alleles found in more than 3 individuals (B). Frequency of MHCI supertypes according to AdV (C) and helminth infection status (D). Frequency of MHCII alleles according to AdV (E) and helminth infection status (F). (TIF) [file ppat.1009675.s003.tif]

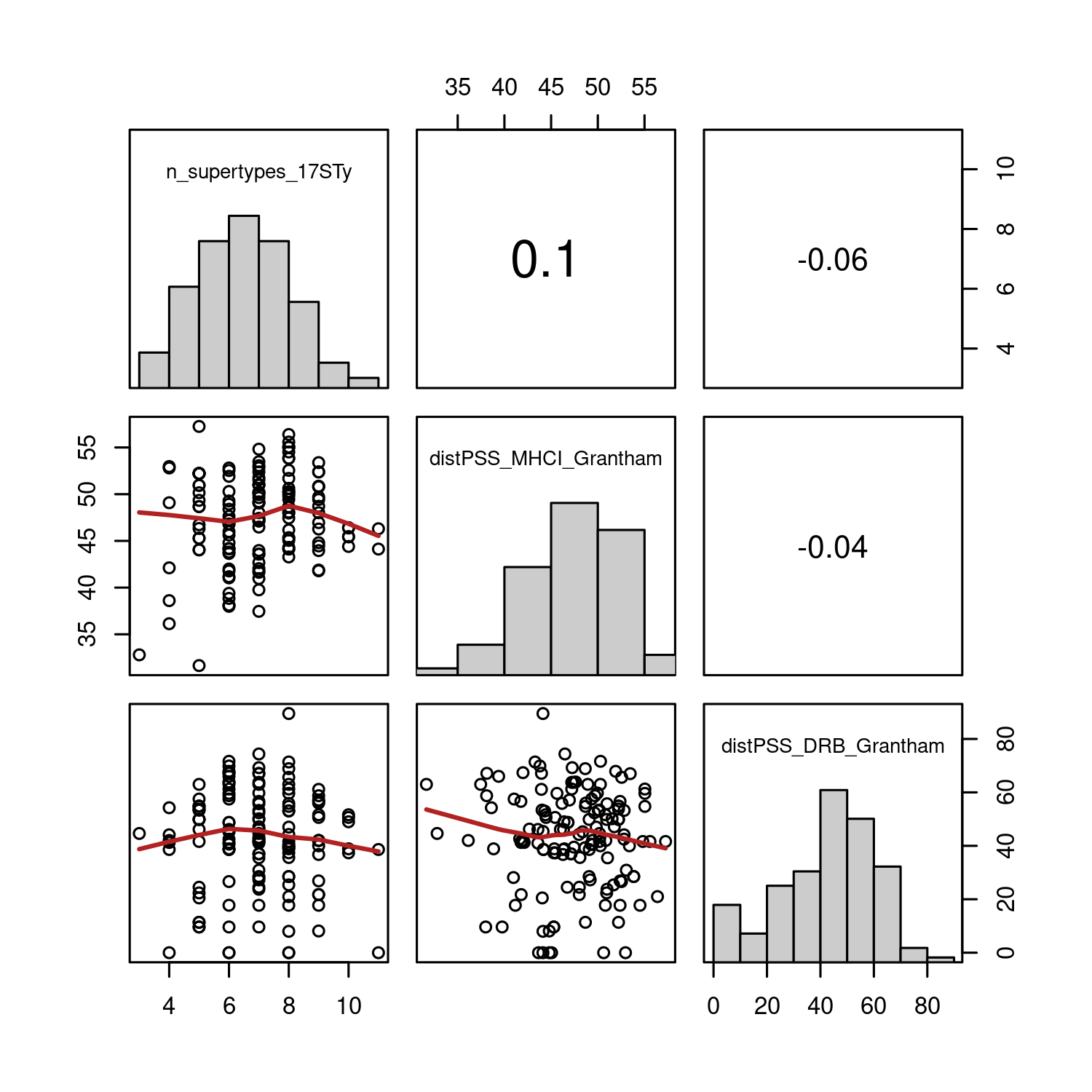

Supplement: S4 Fig — Values within the top diagonal boxes of the correlation plot correspond to Spearman correlation coefficients. (TIF) [file ppat.1009675.s004.tif]

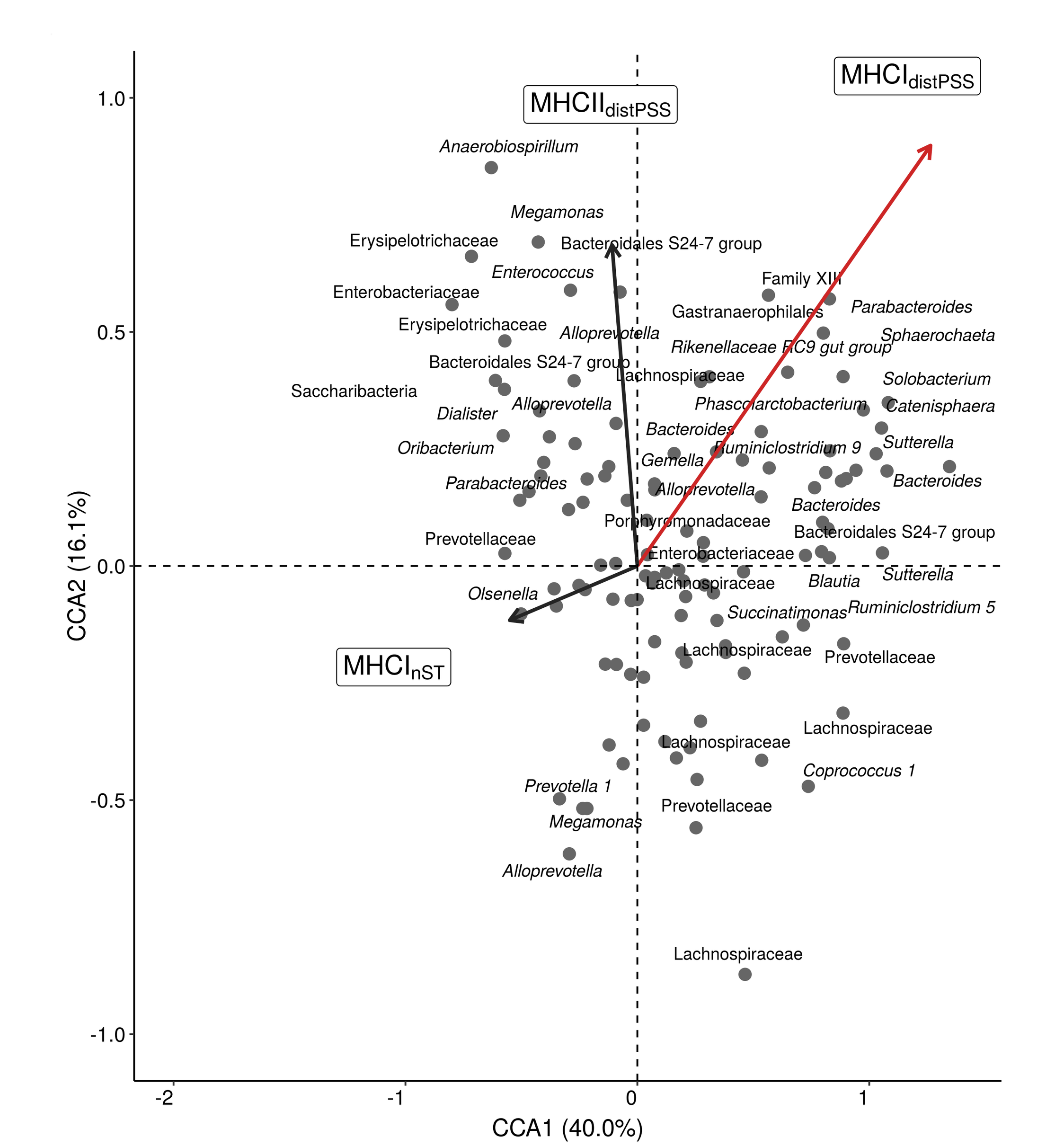

Supplement: S5 Fig — Canonical correspondence analysis (CCA) for taxonomic composition in association with MHC functional diversity (n = 143). CCA biplot depicting ASVs (full circles) and MHC diversity estimates as arrows. MHCIdistPSS (r2 = 0.16, P<0.0001) functional diversity significantly predicting microbiome composition is shown in red. Labels correspond to assigned taxa. (TIF) [file ppat.1009675.s005.tif]

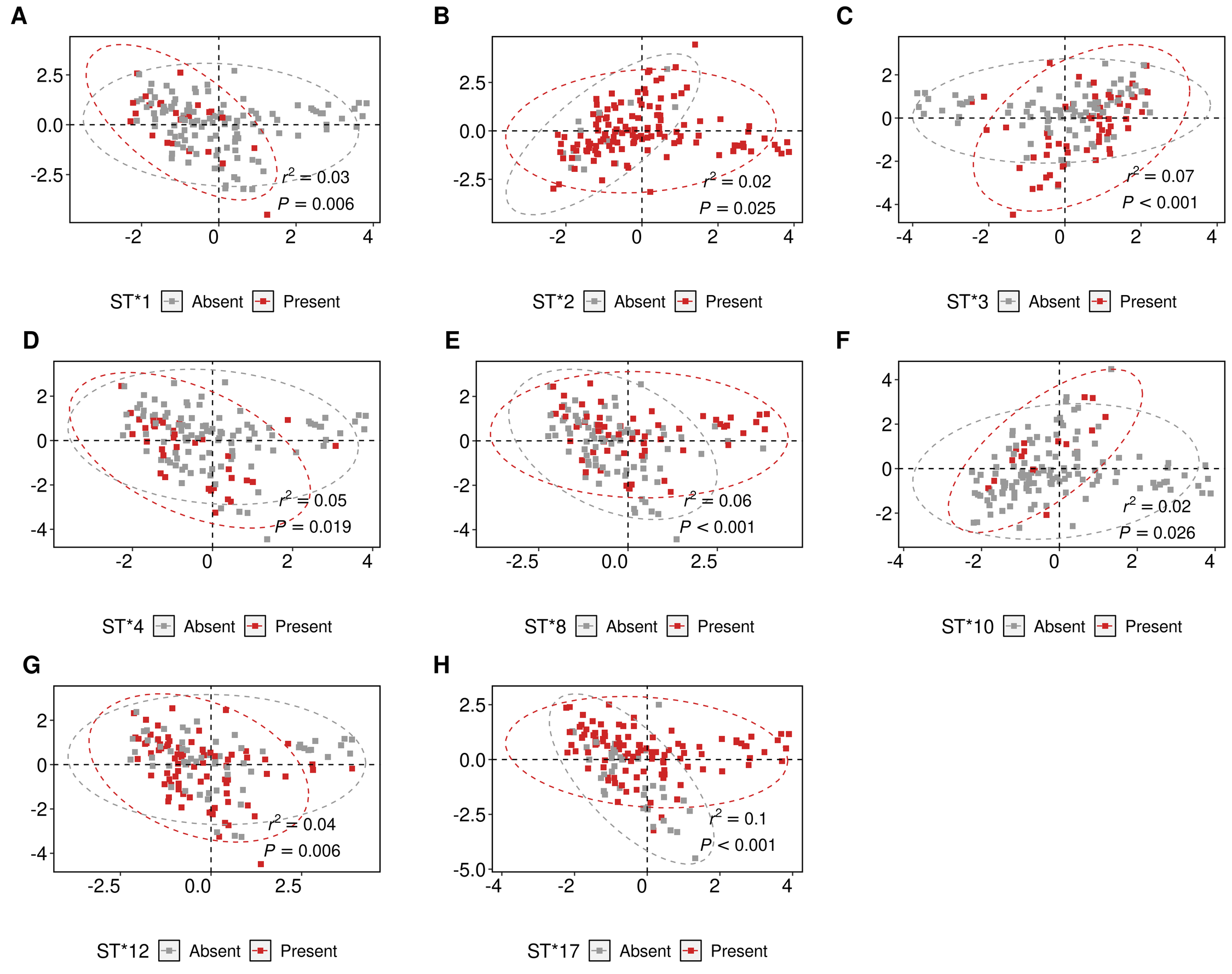

Supplement: S6 Fig — CCA biplot depicting the differences among samples according to the presence/absence of the MHCI supertypes. (TIF) [file ppat.1009675.s006.tif]

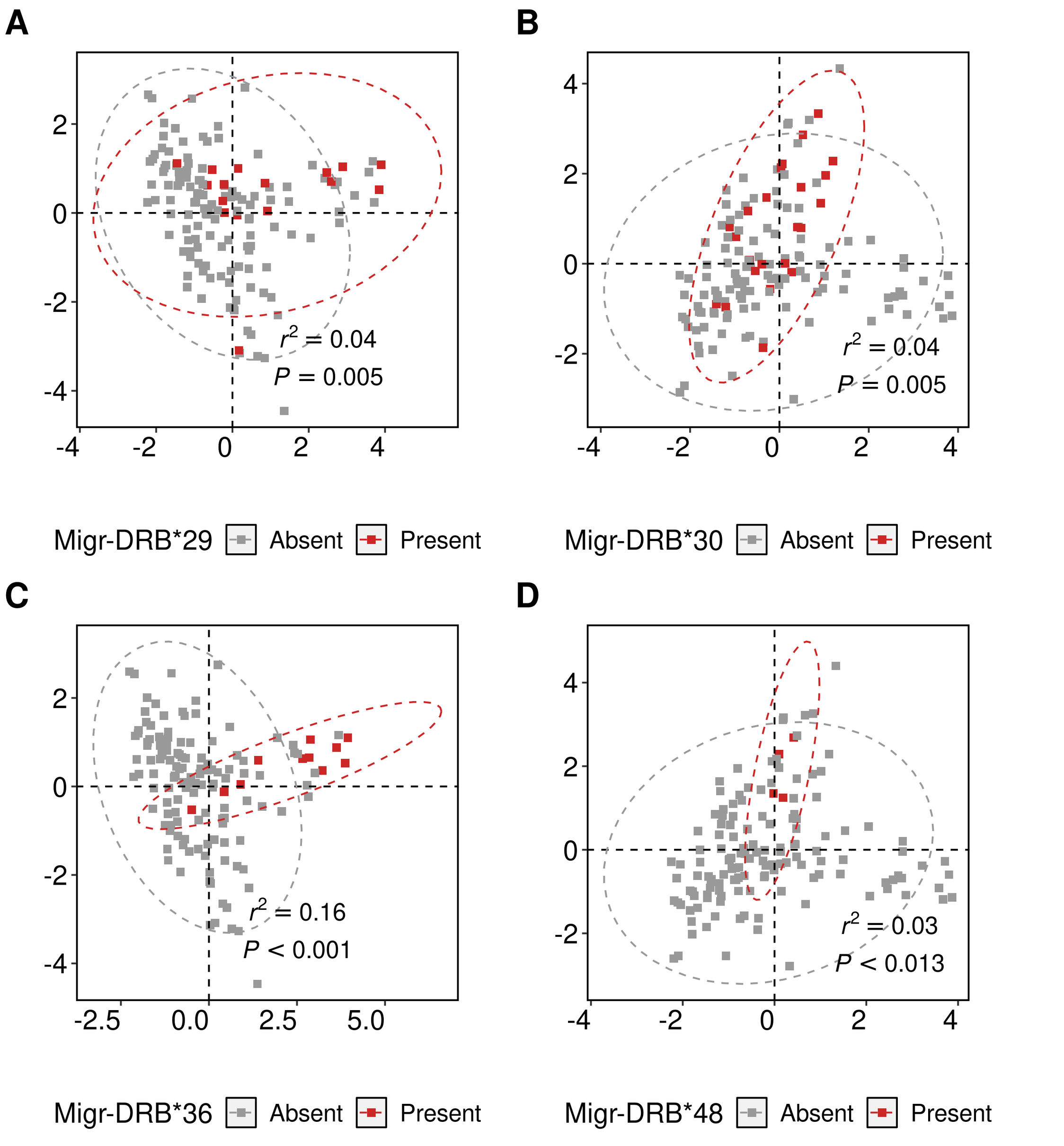

Supplement: S7 Fig — CCA biplot depicting the differences among samples according to the presence/absence of the MHCII alleles. (TIF) [file ppat.1009675.s007.tif]

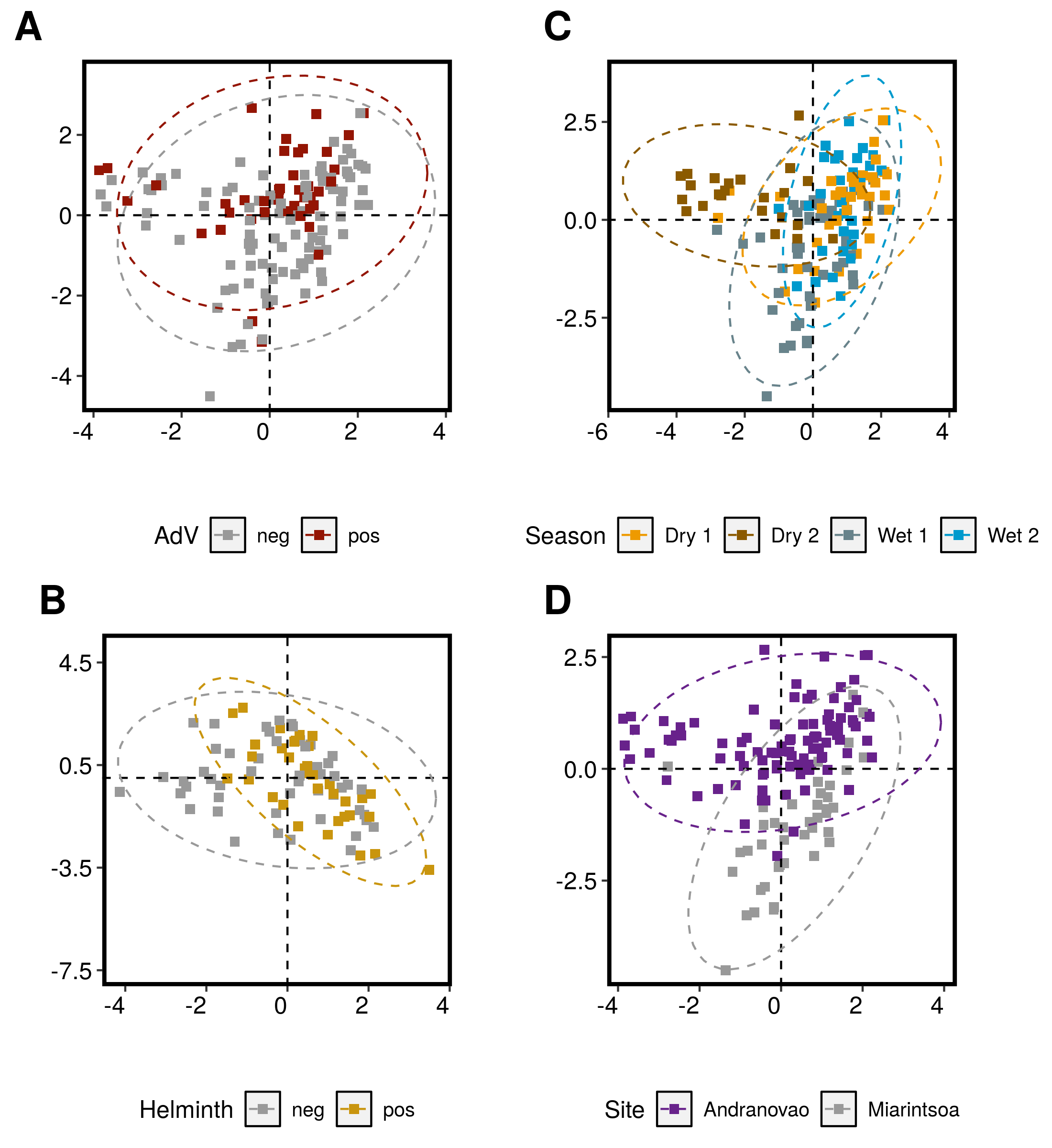

Supplement: S8 Fig — (TIF) [file ppat.1009675.s008.tif]

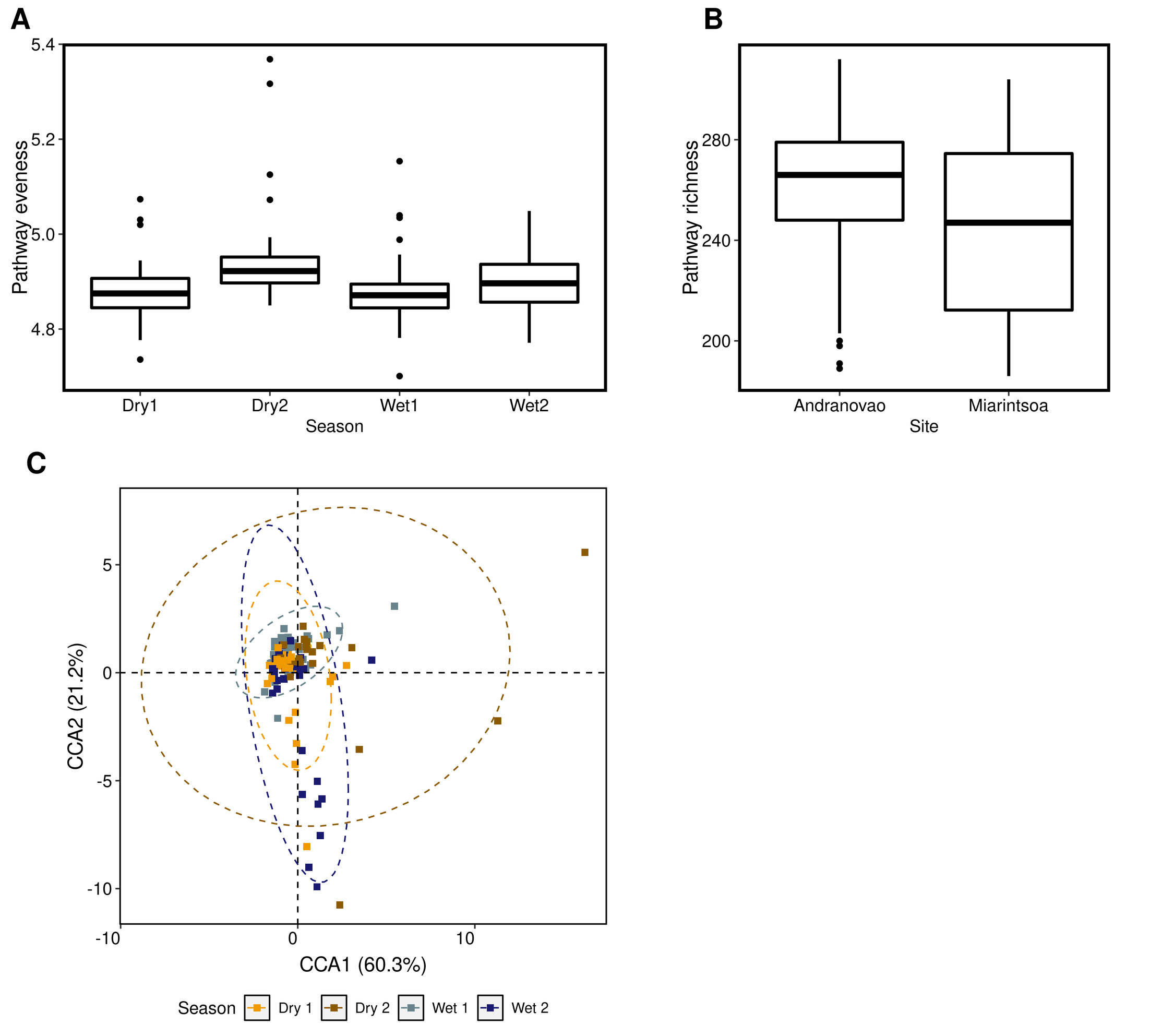

Supplement: S9 Fig — Boxplots illustrating differences in pathway eveness according to season (A), pathway richness according to site (B) and CCA biplot depicting differences among samples according to season (C). (TIF) [file ppat.1009675.s009.tif]

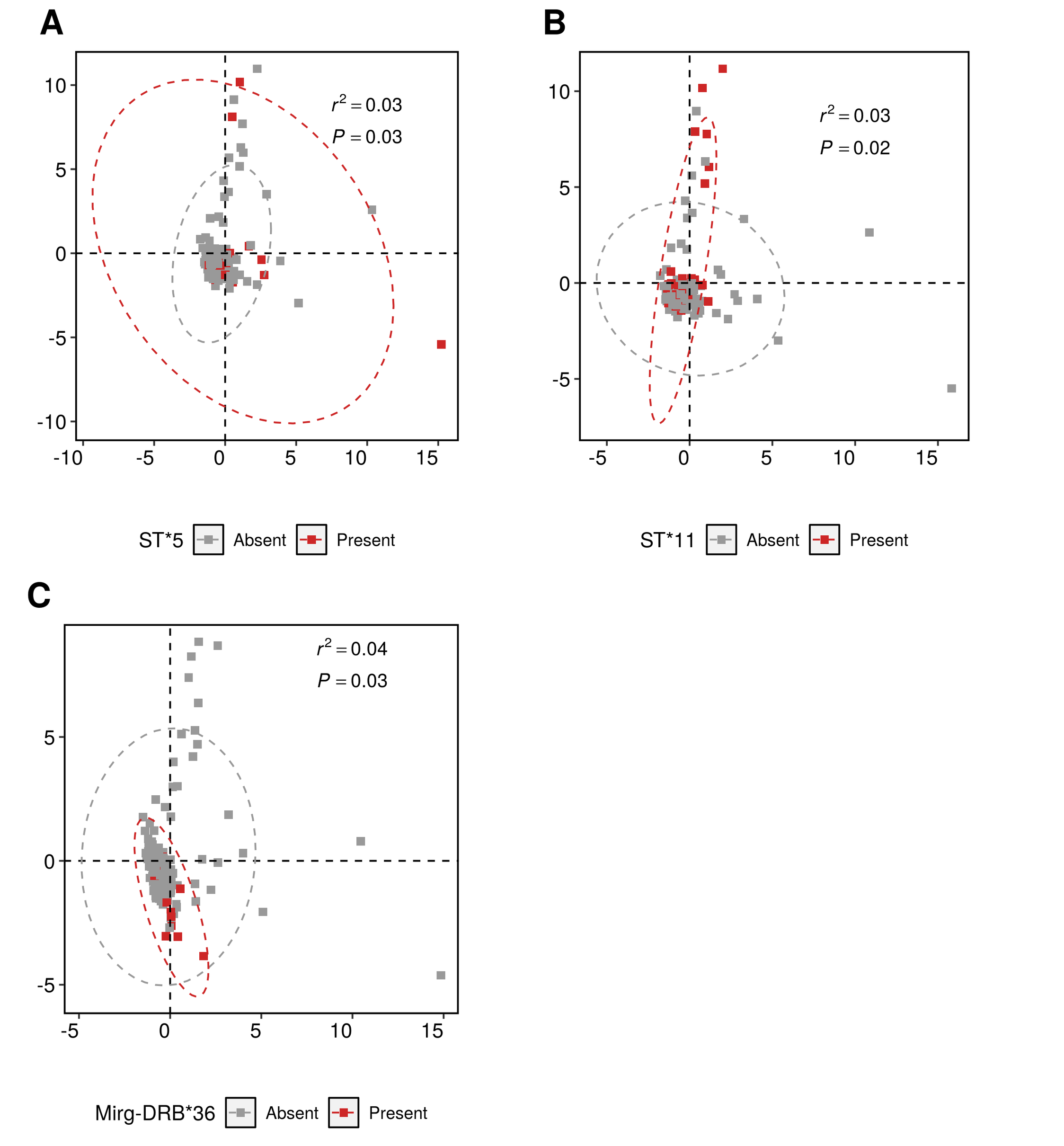

Supplement: S10 Fig — CCA biplot depicting the differences among samples according to the presence/absence of the MHCI supertypes and MHCII alleles. (TIF) [file ppat.1009675.s010.tif]

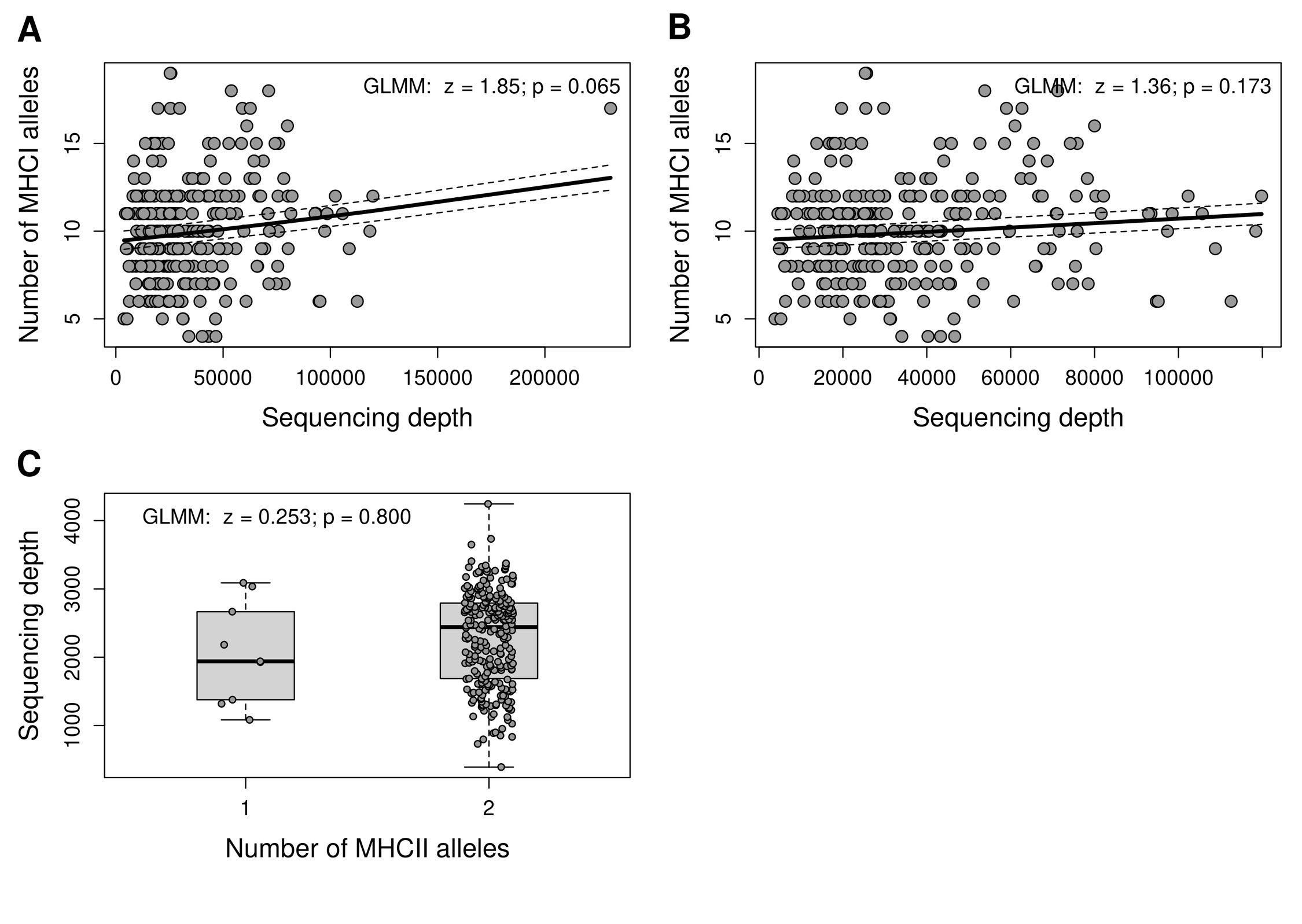

Supplement: S11 Fig — Association between sequencing depth and number of MHCI alleles (A), after removal of an outlier (B), and the number of MHCII alleles (C). Sequencing depth used for these analyses was after denoising, i.e. after removal of chimeras, singletons and non-target sequences according to a BLAST search using the ACACIA pipeline (see Material and methods for pipeline methods). Generalized linear mixed models (GLMM) for MHCI ((A B)) included number of alleles as a response variable, sequencing depth as an explanatory variable and sample ID as a random factor and were fitted with a Poisson distribution with a log link. The GLMM for MHCII ((C)) included number of alleles as a response variable (1 or 2 alleles), sequencing depth as an explanatory variable and sample ID as a random factor and was fitted with a Binomial distribution with a logit link. (TIF) [file ppat.1009675.s011.tif]

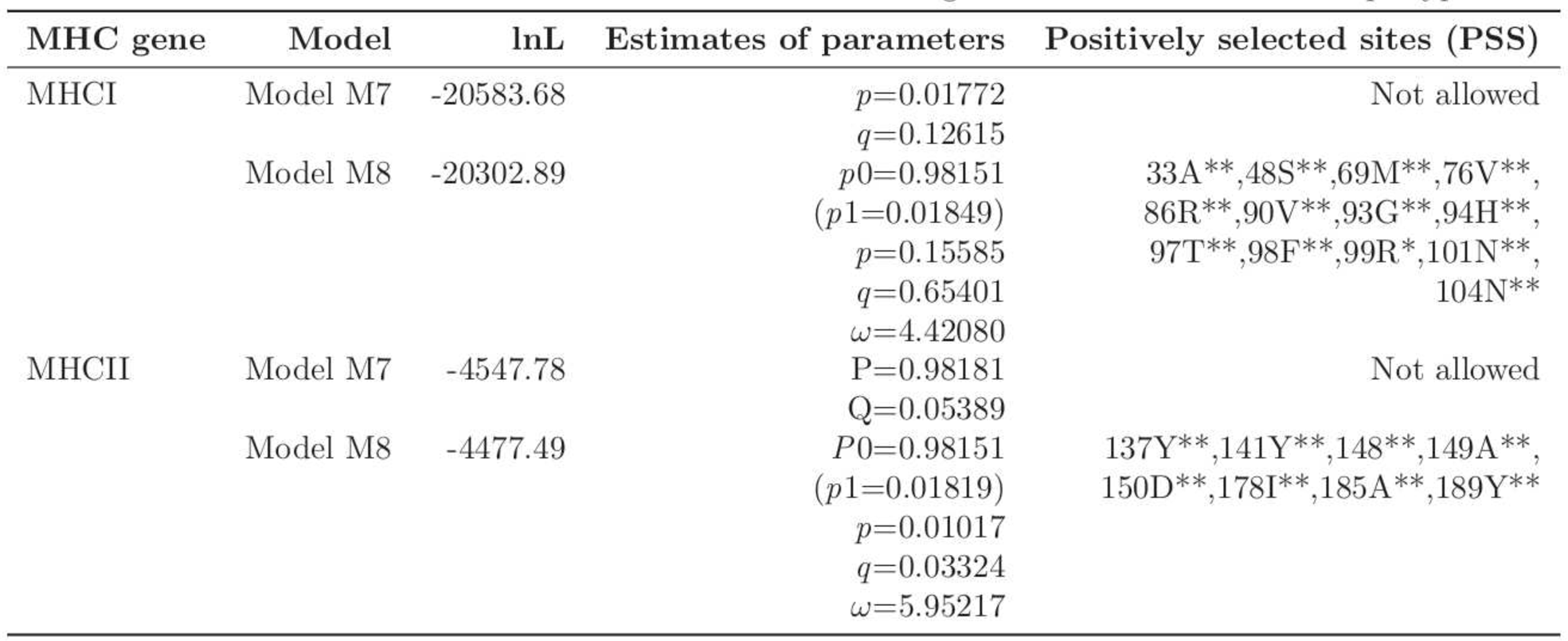

Supplement: S1 Table — Log-likelihood values and parameter estimates of models testing for positive selection acting on MHC class I and MHC class II DRB exon 2 of Microcebus griseorufus. Parameters p and q computed from the beta distribution. ω = dN/dS ratio. pn = of sites that fall into ωn site class. Site positions inferred to be under positive selection estimated at a * 95% and ** 99% confidence interval level. Amino acid numbering is based on the HLA-A2 haplotype. (TIF) [file ppat.1009675.s012.tif]

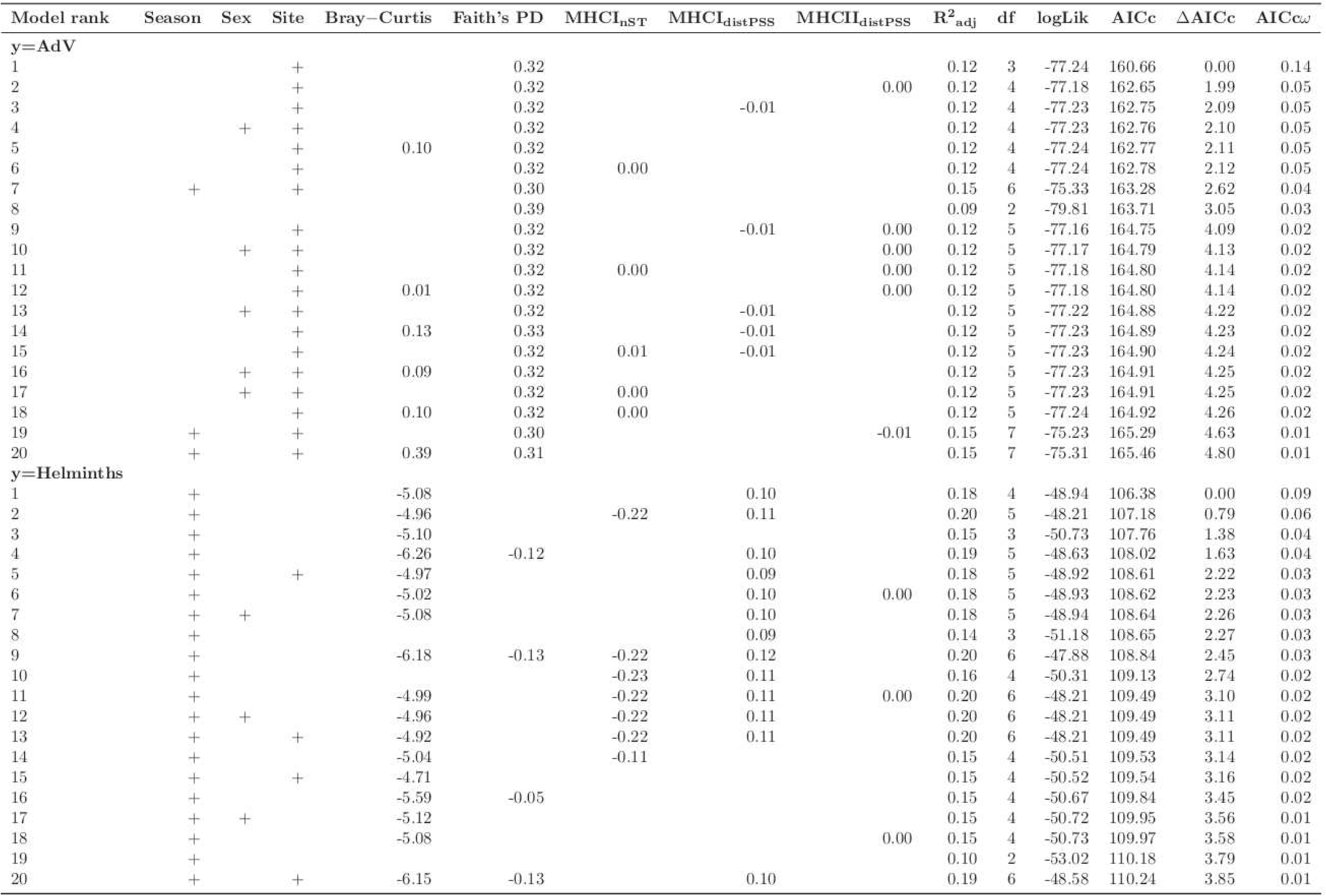

Supplement: S2 Table — Model selection of GLMs (top 20 models subset) examining the association between MHCI and MHCII motifs and diversity estimates, microbiome diversity (Faiths’ PD) and divergence (Bray-Curtis) and covariates on AdV and helminth infection status. AICc = Akaike Information Criterion for small sample sizes; AICcω = AIC weight; “+” denotes categorical parameters included in the model whilst the inclusion of a continuous variables is indicated with its estimate. (TIF) [file ppat.1009675.s013.tif]

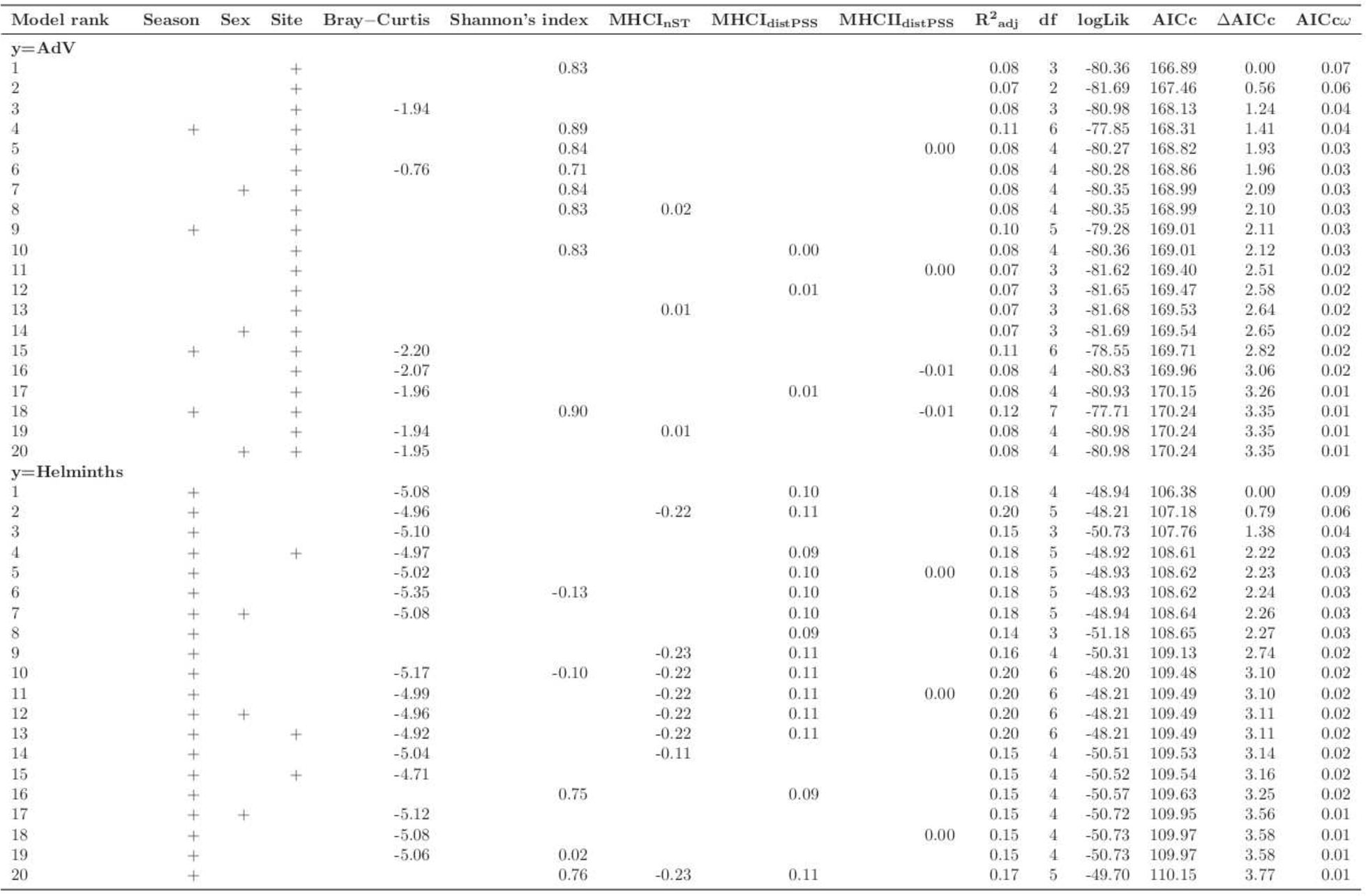

Supplement: S3 Table — Model selection of GLMs (top 20 models subset) examining the association between MHCI and MHCII motifs and diversity estimates, microbiome diversity (Shannon’s index) and divergence (Bray-Curtis) and covariates on AdV and helminth infection status. AICc = Akaike Information Criterion for small sample sizes; AICcω = AIC weight; “+” denotes categorical parameters included in the model whilst the inclusion of a continuous variables is indicated with its estimate. (TIF) [file ppat.1009675.s014.tif]

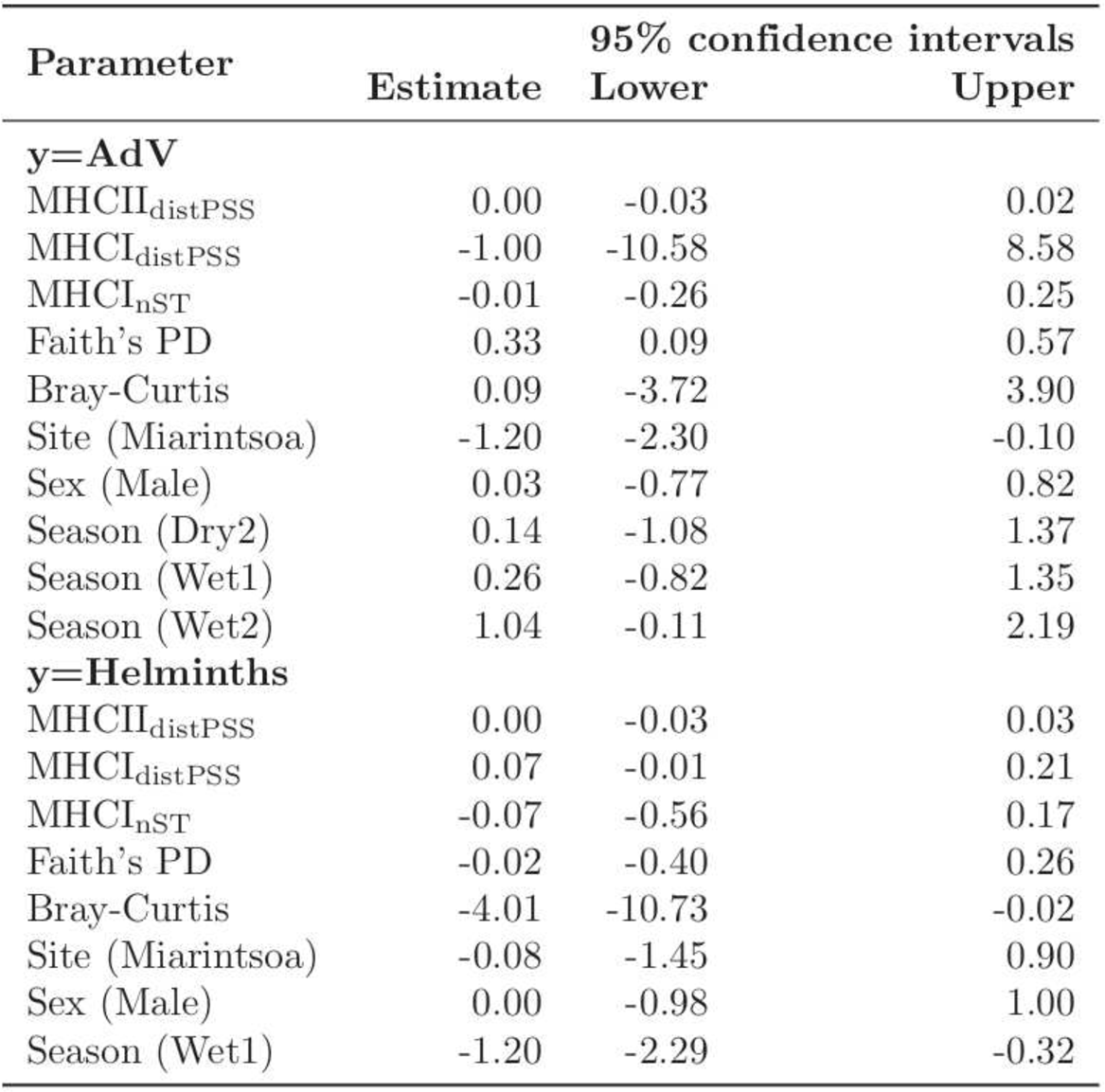

Supplement: S4 Table — (TIF) [file ppat.1009675.s015.tif]

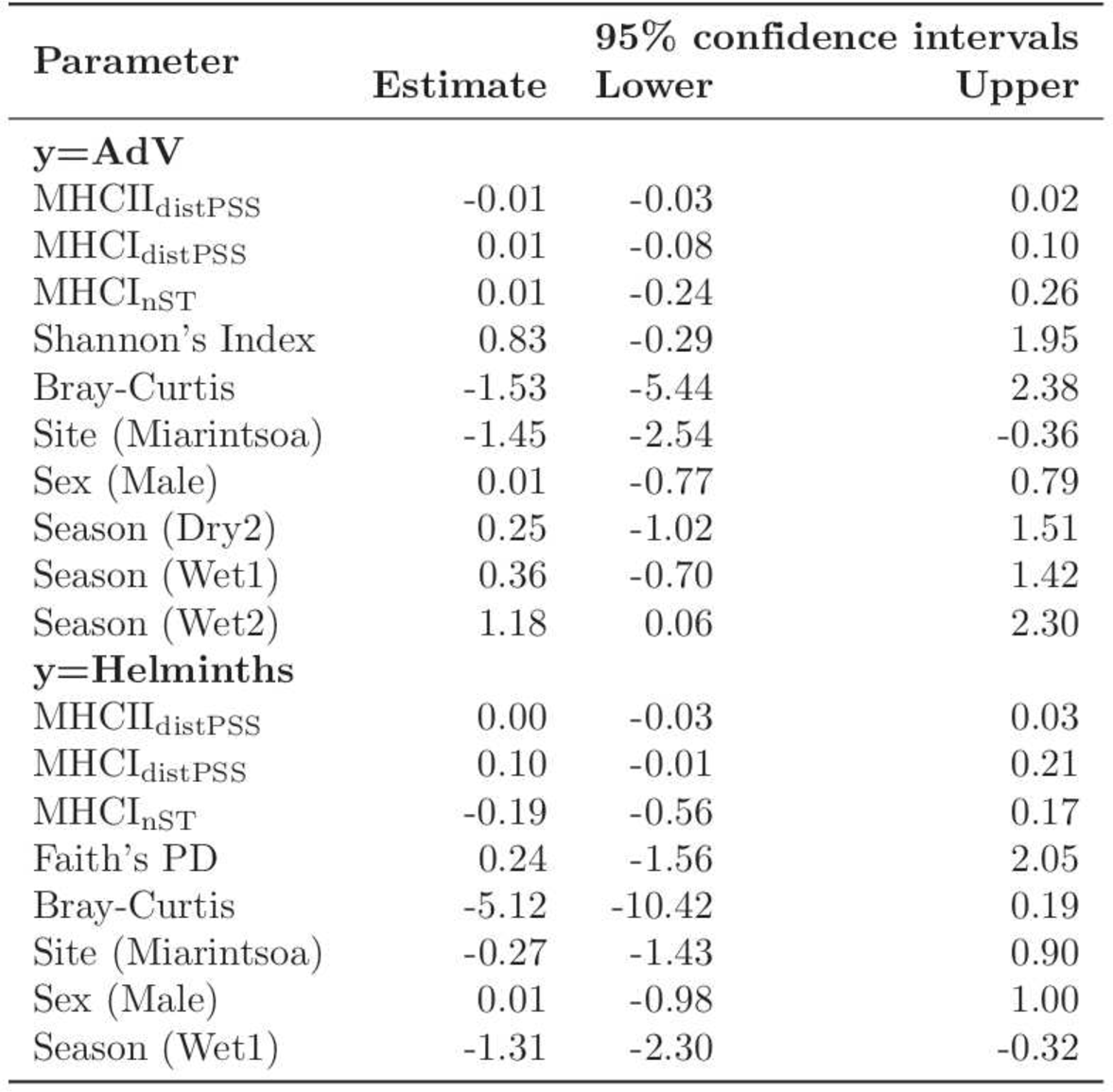

Supplement: S5 Table — (TIF) [file ppat.1009675.s016.tif]

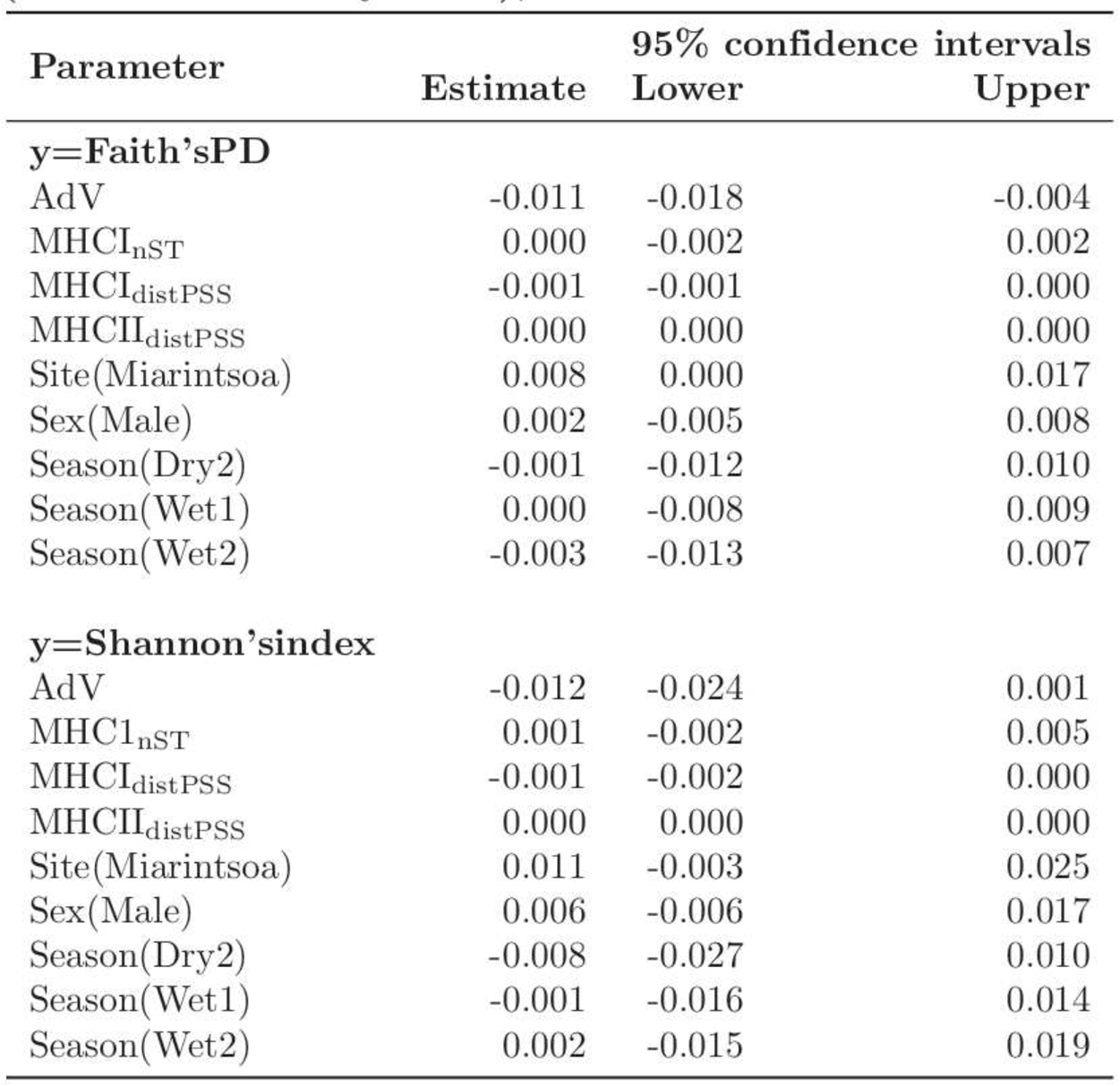

Supplement: S6 Table — (TIF) [file ppat.1009675.s017.tif]

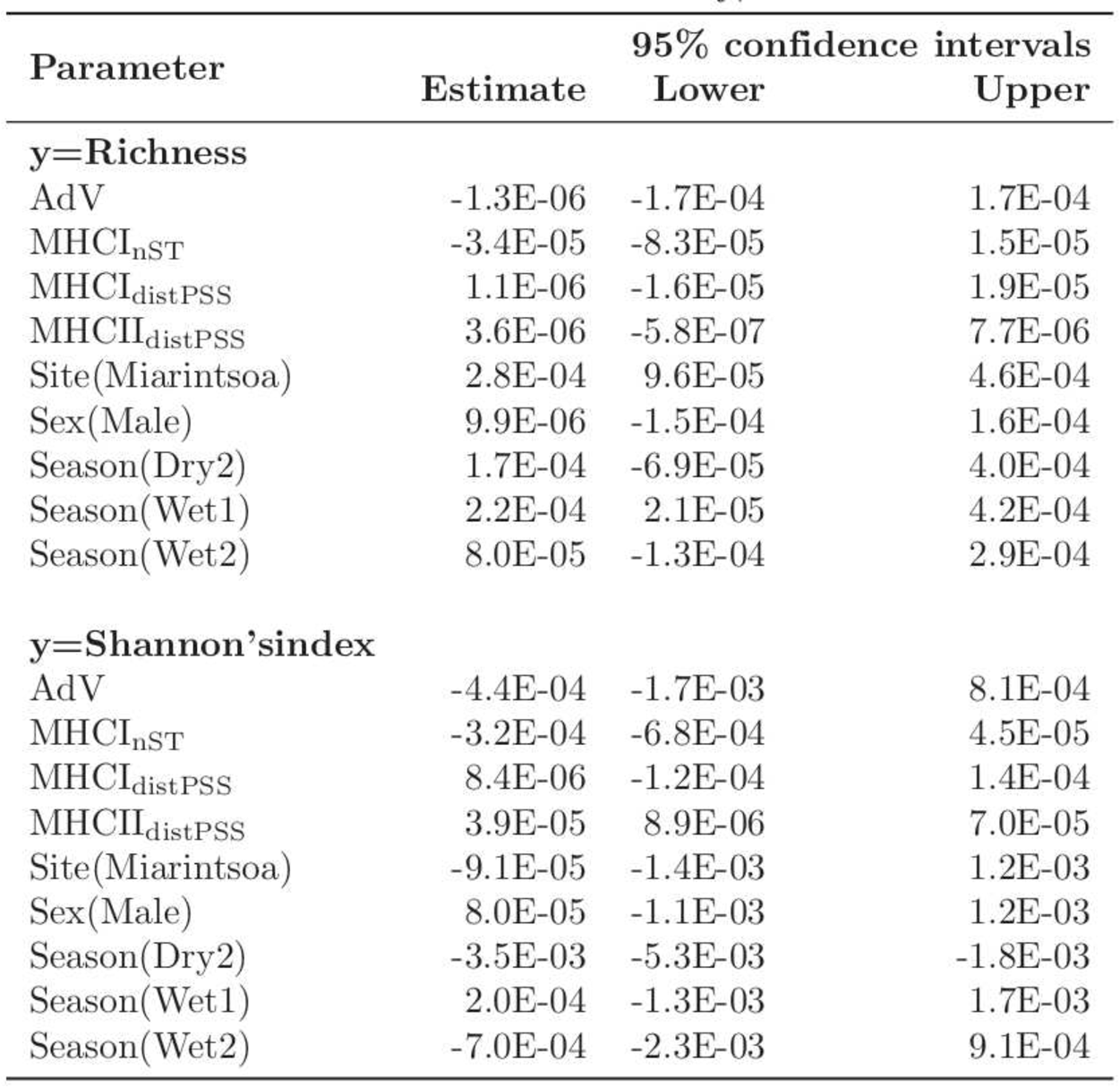

Supplement: S7 Table — (TIF) [file ppat.1009675.s018.tif]

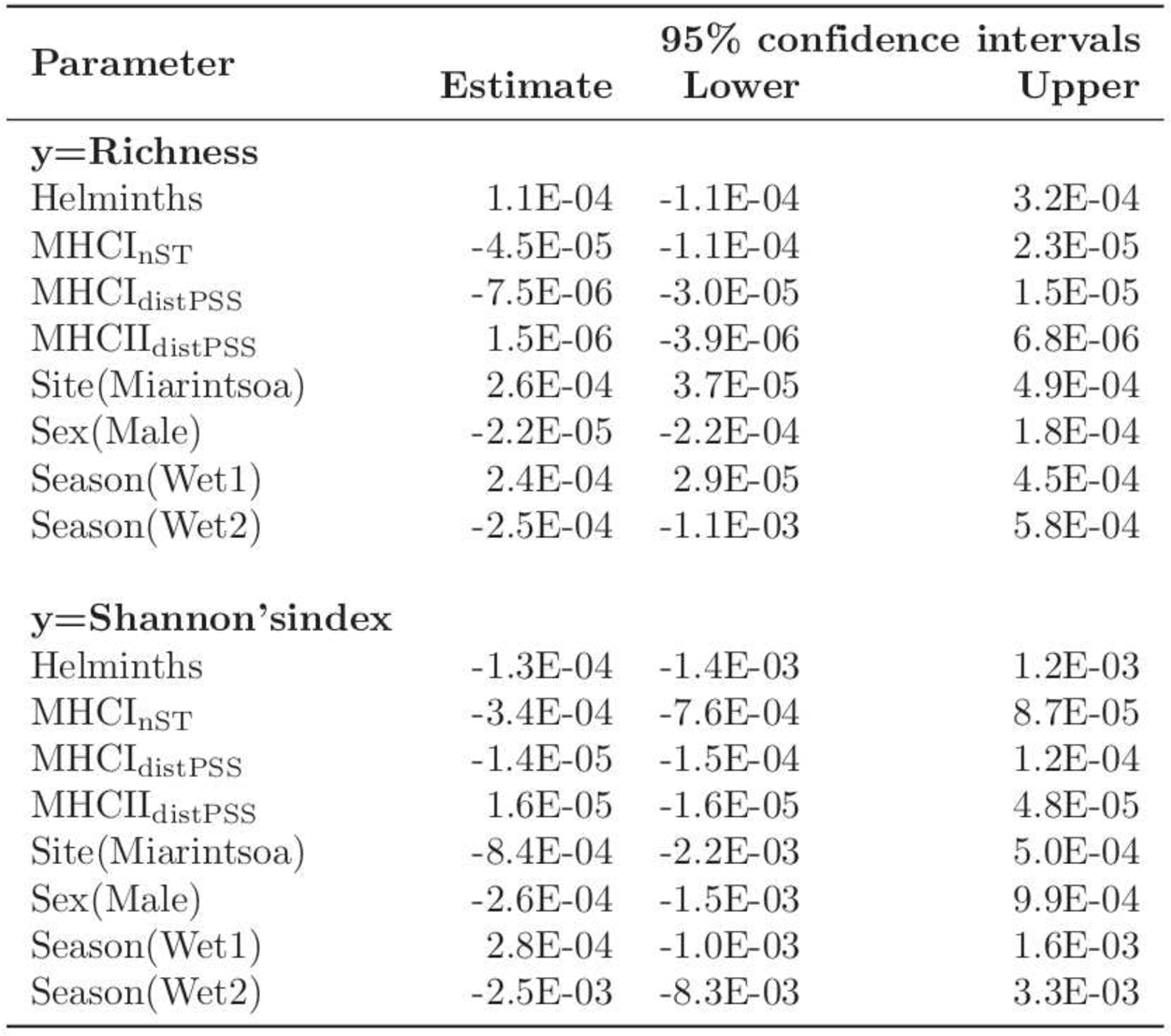

Supplement: S8 Table — (TIF) [file ppat.1009675.s019.tif]
