# Supplementary material for: Evidence of MHC class I and II influencing viral and helminth infection via the microbiome in a non-human primate
Source: PLoS Pathog. 2021 Nov 8;17(11):e1009675. doi: 10.1371/journal.ppat.1009675 (PMC8601626; doi:10.1371/journal.ppat.1009675)
Supplement: Attachment — Submitted filename: Rebuttal_MHC_microbiome.pdf [file ppat.1009675.s020.pdf]

Universität Hamburg
DER FORSCHUNG | DER LEHRE | DER BILDUNG

B. Karina Montero, PhD

B. Karina Montero
Biozentrum Grindel
Martin-Luther-King Platz 3
Hamburg 20146, Germany
Phone: (+49)731 5022641
E-mail: b.karina.montero@gmail.com

August 27, 2021

Editorial board
PLOS Pathogens

Dear Editors,

On behalf of all co-authors I would like to thank you and the three reviewers for the constructive comments and suggestions. We considered them all and revised the manuscript accordingly. We would like to resubmit the revised version of our manuscript "**Evidence of MHC class I and II influencing viral and helminth infection via the microbiome in a non−human primate**".

We are very grateful for the positive and highly constructive comments from the reviewers and to the editors for the opportunity to resubmit a significantly revised version of the manuscript addressing the concerns raised. As a summary of our major changes in this revised version, we have simplified the presentation of our analyses, clarified the effect of sampling site and more carefully interpreted and accounted for the correlative nature of our study (including with regards to linkage with other genes within the MHC region and MHC supertypes). Reflecting these major changes, the current version of the manuscript contains a new figure in the main text (Figure 2) as well as revised and new figures in the supplementary material (Figure S3, S4, and S11). Moreover, we have also ensured that both the data (raw data and sequence accession numbers) and the analytical approach (R scripts) used in this study is publicly available to all readers.

We believe that this review process has greatly improved our manuscript and we wish to thank again the reviewers and editors for their thoughtful and constructive comments. We hope that this heavily revised version addresses all of the concerns raised by the reviewers and that this study meets the standards of publication in *PLOS Pathogens*.

Please find the detailed responses to each reviewer's comments below. All responses are marked in blue. We provide a version of the manuscript that tracks the changes made to the text. In the track-changed manuscript all changes are marked in blue.

Sincerely on behalf of all coauthors,

B. Karina Montero, PhD

Reviewer #1

Part I – Summary

The study focuses on an area of tremendous interest; the contribution of host genetic variability, particularly within the major histocompatibility complex, in shaping the composition of the gut microbial community. The premise is particularly important and the authors combine modern laboratory techniques with field work, which is both novel and highly interesting.

Answer: We are very grateful for the very positive comments from the reviewer which we feel substantially improved our manuscript. We hope that we have now addressed all of the concerns raised.

However, I have serious concerns regarding the interpretation and presentation of the data (e.g., an animal is either AdV+ or AdV-; your assay either detected AdV or it didn't. There is no reason to present a per-individual probability of infection stratified by MHC status. Either the MHC group is enriched for AdV+ or AdV- individuals or it isn't.). All such oddities need to be corrected and better communicated. No modeling is necessary to test the hypothesis that AdV-positivity segregates with DRB*34-positivity. Keep it simple.

Answer: We agree with the reviewer that the presentation of the results can be simplified. To address the reviewers' comment, we modified our statistical approach in two ways. First, we simplified our analytical approach to examine the relationship between infection status and the presence of MHC motifs by presenting the results from odds-ratio tests and Bonferroni adjustment for multiple comparisons (this replaces the probabilistic approach using co-occurrence analysis). To support this simple analysis, we provide a modified version of Fig. S4 showing the frequency of AdV and helminth infected vs non-infected individuals according to MHC motif (i.e. raw data of infection frequencies according to MHC motifs). Second, we have removed the effect of Migr-DRB*34 and ST*3 from the generalized linear models (GLMs) from the results and the discussion. Removing these terms from our GLMs led to increased model support for the effect of site on infection status. This effect is now reported in Supplementary Tables S2-S5 and plotted in Figure 2 A and B. Also, to clarify the type of infection data used in our study (i.e. infection status) we made the following modifications to the text:

- Introduction, **L 46-47**: Changed "To this end, we explored adenovirus (AdV) and helminth infection probabilities" to "To this end, we explored adenovirus (AdV) and helminth infection status".
- Figure 2 legend: Replaced "infection probability" with "infection status".

Samples were collected from the same species of lemur at two distinct sites and at different time periods (i.e., different seasons), which differ significantly with regard to variety of parameters that could influence the composition of the gut flora. More critically, the populations at the different sites may be genetically distinct, but there is no presentation of data or discussion of results that indicate that the authors thoughtfully partitioned their datasets to determine if their analyses were biased by site (I suspect that they are, but how could I know– they only state "we evaluated... while controlling for extrinsic factors (sampling site and season)" and "we used binomial GLMs to examine the effect of MHC and microbiome diversity on parasite infection while controlling for the potential effects of covariates (site, season, and sex)." That is pretty all encompassing. I want to see the raw data.

Answer: We completely agree with the reviewer that it is crucial to account for the effect of confounding variables associated to our sampling design. For this purpose, we consistently included sex, study site and season as covariates throughout our statistical analysis and report their effect. For instance, the effect of site and season on microbiome taxonomic composition and functional pathways is reported in lines 134-140 and lines 208-215, respectively. Figures supporting these results are provided in the Supplementary Material (Figure S8 and S9). In

addition, following the reviewer's suggestions (please see above), our revised analyses revealed a better supported effect of site on infection. Consequently, we hope that the effect of covariates such as site is clearer in the current version of the manuscript, since we provide a new figure of the effect of site on AdV infection status (Figures 2 A and B).

The authors assume that once infected, AdV infection is persistent. Yet, there is no discussion of this reality that AdV infection can be transient and therefore, particularly without any immunological data, it is impossible to know the true relationship between host genetics and susceptibility to infection. The authors clearly have the ability to address this, and so they should.

Answer: As pointed out by the reviewer, our study does not provide evidence of persistent AdV infection on *Microcebus griseorufus*. Even though it was not our intention to analyze and interpret our data as a persistent infection, we realize that the following statement in the Introduction might be misleading: "Chronic viral infection, helminths and commensal bacteria share the common agenda of promoting acceptance by its host, often via immune regulation.". Therefore, to address the reviewer's comment we modified the text to clarify the fact that our study is focused on AdV infection status at the time of sampling: "Viral infection, helminths and commensal bacteria share the common agenda of promoting acceptance by its host, often via immune regulation.". Throughout the remaining sections of the manuscript, we ensure that we do not interpret viral infection as a chronic infection but as infection at the time of sampling. Finally, we added the following sentence: "We emphasize that our data reflects punctual infection status rather persistent infection." (lines 473-474) in the methods to emphasize to the readers that our data reflects punctual infection and not persistent infection.

The authors need to show that the MHC composition gut flora does not differ by site if they are going to combine the two populations of animals.

Answer: We understand the reviewers' concern to account for the effect of site (and other covariates) in our analyses. As mentioned above the effect of site is accounted for statistically across all analyses. We found support for an effect of site on microbiome phylogenetic diversity but not on eveness (taxonomic features: Table S2-S5; metabolic pathways: Table S8). Furthermore, the effect of site and season on microbiome taxonomic composition is reported in Fig. S8, and their effect on metabolic pathways is reported in Fig. S9. We discuss the effect of site and season on microbial composition in lines 134-140 and lines 208-215 and to our knowledge we have taken every precaution to statistically account for their effects. We hope that in this new version of the manuscript the changes mentioned above now make the effect of site much clearer to the reader and the reviewer.

This manuscript needs significant revision; not necessarily new work, but potentially re-analysis to simplify the substantiation of the authors' largest claim. It is otherwise an important topic that few groups are positioned to investigate, and this group is clearly well positioned.

Answer: We again thank the reviewer for their positive comments and for their support. We hope we have now addressed all of the issues raised including the simplification of some analyses and their presentation.

Part II – Major Issues: Key Experiments Required for Acceptance

Simplify your analyses where possible. This is a major generalization, but the hypotheses is much simpler than is presented: "MHC genotype influences infection susceptibility to X" and "MHC genotype influences the composition of the gut microbial communIty."

Answer: We have addressed this comment in detail above (please see the first answer). We now hope that the issues raised by the reviewer has been satisfactorily addressed in this new version of the manuscript.

The authors need to show that AdV-negative animals are sero-negative as well and they need to control for animal age. The same needs to be done for Helminth. . . they make a

major assumption that once infected, always infected, and it isn't entirely clear that this is the case.Otherwise, they need to reconstruct their premise.

Answer: The reviewer raises an important point however, it is crucial to bear in mind that AdV, helminth infection, and the gut microbiome is assessed non-invasively (please see Materials and methods section, lines 444-463) through punctual sampling of faeces whilst the MHC was genotyped using ear tissue samples (the latter is also less invasive compared to blood samples). Since we are working with a primate species, non-invasive sampling is prioritised. As there is no blood we can't investigate the antibody status of these animals and can't say that they are sero-negative. We would also like to note that even if blood was available for parasite screening, building up an antibody detection tools, particularly when working with a wildlife species, is not a trivial task. Nonetheless, as mentioned above, we agree with the reviewer that by using the term "chronic infection" and by not clearly stating what kind of parasite infection data we used for our study might cause confusion to the reader. Therefore, we have now removed the word "chronic" and we ensured that all data interpretation concerns infection status at the time of sampling. In addition, we added the following sentence: "We emphasize that our data reflects punctual infection status rather persistent infection." (lines 473-474) in the Materials and methods section to emphasize that data only reflects punctual infection.

Concerning the reviewer's comment to include age as a covariate in our analyses, there are two issues that prevent us from directly controlling for age. First, it is notoriously difficult to accurately estimate age of mouse lemurs (see Radespiel *et. al.* 2019. American Journal of Primatology). Indeed, fast development of offspring to reproducing juveniles (this can occur from one rainy season to the subsequent dry season) results in an overlap in the presence of young and older adults that cannot be reliably distinguished by their outer appearance. Second, the best proxy of age is weight, however this indirect estimate is collinear with season which is included in all of our statistical analyses. Therefore we cannot include both season and age (which can only be indirectly estimated anyways) in the same model and we chose to prioritise season which is a more ecologically relevant parameter for our study. However, using age instead of season does not substantially change the results of our study and results in a loss power since using a weight threshold as a proxy of age results in the exclusion of some individuals where age could not be confidently estimated (approx. 20 individuals from a total of 143 or 14% of the dataset). To address the reviewer's comment, we added a statement in the Materials and methods section that clarifies why in our study system age is not included (lines 576-580).

You state that 29.4% of individuals = 143 animals on line 57. You also state that 40.1% of individuals = 86 animals on 57-58. While I understand that you are referring to percentages for subsets, the way it is presented is confusing.

Answer: We understand that the fact that we have two subsets may be rather confusing to readers. In this new version of the article we attempted to clarify this by adding in brackets the total number of individuals that tested positive as well the total number of individuals tested. See lines 67-71.

Line 86 and 93, you refer to the wrong figures.

Answer: Thanks! We have checked that all Figures and Tables are cited in the appropriate place.

Reviewer #2

Part I – Summary

This manuscript describes a project to correlate information about MHC class I and class II sequences, bacteria in the intestinal microbiome, helminth carriage and adenovirus infection using a 124 individuals of a wild mouse lemur colony. The data collection alone must have been an enormous effort, and then a wide variety of statistical analyses were applied to look for correlations. I am not qualified to evaluate the model building and statistics, but I know a fair amount about the MHC, and a bit about viruses, bacteria, helminth parasites and immune responses. Overall, the authors are to be commended on an outstanding paper with very sensible approaches, but I do have a few concerns which the authors might do well to consider.

Answer: We are very grateful for Prof. Jim Kaufman's highly constructive comments and for his very positive feedback. We hope that we have addressed the concerns raised below.

Perhaps my major concern is the assumption that the MHC class I and class II genes that are typed by sequencing and used for the correlations are responsible for all the biological effects. The MHC in general is a large region with many different kinds of genes, and there are many examples of traits initially ascribed to classical class I and class II genes that turned out later to be due to linked genes with entirely different functions. Just one very recent example is the importance of C4 alleles on sex-linked diseases including various autoimmune syndromes and schizophrenia (Kamitaki et al 2020 Nature). However, it is not just the genes involved in immunity such as the complement components factor B, C2 and C4 and the cytokines TNF and lymphotoxins, but genes that might affect any aspect of physiology, cell cycle and the like, given that it is complex biology being examined. It is easy to imagine a cell cycle or TRIM gene (located near a particular class II allele) with a point mutation in the coding region or in the promoter/enhancer to affect adenovirus resistance. It is even plausible that all the class I genes of a particular supertype have a common ancestor, so that the surrounding haplotypes of all those class I alleles contain an allele of C4, TNF or steroid hydroxylase that affects helminths. The careful interpretations and discussions throughout the manuscript do not need to be re-written, but near the beginning and at the end, the authors should consider these alternatives, which afford a wider view of the possibilities.

Answer: We agree with this comment and that it was an important omission on our part. Many thanks for the detailed suggestion on how to address this issue. In this new version of the article we have followed the above suggestions and we have included a new sentence in the Introduction section (lines 18-27) and a new paragraph in the discussion (lines 370-379). In the introduction we reminded readers (and ourselves ;)) that the MHC is indeed a complex genomic region with a large number of genes in linkage disequilibrium with the classical MHC genes that we focus on in this study. We are also very grateful for the provided insightful example to illustrate the importance of this point (the study from Kamitaki et al 2020 Nature). Given its relevance we now used this same example in the current version of the discussion. In the added paragraph in the discussion, we recognise that the correlations between MHC I and II diversity can also be attributed to other genes in linkage disequilibrium.

A second concern is that the authors have chosen to lump class I alleles together as supertypes. I am aware of the long and laudable history of simplifying the complexities of human MHC alleles by combining closely-related alleles together, the need to reduce the number of alleles to achieve significance in epidemiology, and the fact that there are no data about actual peptide binding for most non-model organisms. However, there are ample data to show that closely-related alleles can have enormously different effects: consider the many subtypes of HLA-B27 for ankylosing spondylitis (predisposing *2705 and protective *2709 differ by a single amino acid!), or the subtypes of HLA-DRB1*04 which are either strongly predisposing or protective for rheumatoid arthritis (protective *0402 and predisposing *0401/*0404 differ by three amino acids, one of which is key). Moreover, supertypes do not correlate with the size

of peptide repertoire (see for instance Kaufman 2021 Frontiers Immunol), which has recently emerged as an important determinant of resistance and susceptibility to infectious pathogens (summarised in one of the papers cited by the authors, Kaufman 2018 Trends Immunol, and given further support for HIV progression by Bashirova et al 2020 PNAS). Again, the authors do not necessarily need additional experiments or extensive revisions, but these issues should be discussed.

Answer: Many thanks for raising this point. Although in our previous version of the manuscript we did not acknowledge the caveats of using the MHCI supertype we agree with Prof. Kaufman that this is an important issue. In the current version of the manuscript, we provide an in-depth discussion of the potential limitations of using a supertype approach in two different sections of the Discussion (lines 273-285 and lines 326-335). In the first section (lines 273-285), we explain that in our study (and as also mentioned above by Prof. Kaufman) the clustering of supertypes was necessary to achieve our goals given the high allelic diversity found for the MHCI gene. One possible explanation for the lack of an association between MHCI supertypes and parasite infection status may be the consequence of low resolution when working with supertypes (since different alleles within a supertype may have opposing effects in terms of susceptibility to a parasite). In the second section (lines 328-332), we note that the number of MHCI supertypes may be a poor proxy of functional diversity compared to sequence divergence, for the reason mentioned by Prof. Kaufman above. Finally, we mention in our discussion that we only genotyped the DRB of MHCII (line 338). We hope that we have now satisfactorily addressed this concern in this new version of the manuscript.

A third concern is that the authors have apparently analysed a wide variety of class I genes, but focused only on the DRB family of class II genes. I could find at least one other pair of class II genes in the mouse lemur genome on ENSEMBL (likely to be DQA and DQB). What is the evidence that other kinds of class II genes (DQ-like and perhaps DP-like) are not important? Again, this needs to be discussed.

Answer: Prof. Kaufman raises an important point and we agree that it is important to mention why a single gene of the MHCII was genotyped. In our system there is a single copy of DRB, DQA and DQB. All genes are tightly linked, with no recombination hotspots (as opposed to HLA-DQA and HLA-DQB that are separated by a recombination hotspot) and on the same chromosome 6 (DRB and DQB are separated by 100kb with DQA lying in between these two genes). Given that we were operating with a limited budget, and that tight linkage would limit our ability to distinguish whether effects were driven by a specific gene anyways, we prioritised the gene with the highest number of positive selected sites according to a maximum likelihood approach (Huchard et al. 2012. Immunogenetics). We have now added a section in our Material and Methods explaining the reasoning of this strategy (lines 509-513).

A final major concern is the provision of data, rather than just presenting the interpretations of statistical analyses. Unless the authors have a strong reason for withholding such information, the sequences of the alleles (along with their assignment to supertypes for class I) is crucial for readers to understand the paper in more than a superficial way. Also, it is important to include a list of all the individuals examined, with the assignments of MHC alleles along with the presence of adenovirus, helminths and the various bacterial species in the microbiome. The data for the adenovirus and helminths could be presented as presence/absence, or better as MiSeq reads for adenovirus and egg counts for helminths. All this data could be easily presented as a few spreadsheets in supplementary files. The provision of original data is now routine for many biomedical journals and clinical studies, which allows others to examine the same data for validation and for additional research, adding much value to the original paper.

Answer: We understand Prof. Kaufman's concern and we apologize for this. During the submission process we had filled out a form that mentioned that all data and scripts associated to the study would be made publicly available, however we unfortunately did not mention this fact in the main body of the text. In this new version of the manuscript, we added a section

(lines 653-658) to the Materials and Methods that states that all data (including metadata, raw data for infection status of AdV and helminths, the MHC sequences and genotypes, and the raw input files for microbiome analyses in R) and R code scripts are publicly available under figshare (URL links are also provided). We also provide the Genebank accession codes for MHCI and MHCII alleles, and accession IDs of the microbiome sequencing data.

Part II – Major Issues: Key Experiments Required for Acceptance

I did not find the need for additional experiments to support this study, only additional presentation (sequences of class I and class II alleles, list of individuals with MHC type along with presence/absence of adenovirus, helminths and various bacterial taxa of the microbiome).

Answer: As mentioned above this is now done.

Part III – Minor Issues: Editorial and Data Presentation Modifications

Page 3, legend to Fig. 1. "... we predicted that MHC I and MHC II variability influences the diversity and the composition of the microbiome." I was pleased to see that the authors defined the words motifs, diversity and divergence for MHC (which are used in different ways by different people) in terms of their assays, but in this phrase they introduce the term "variability". What do they mean by that, and how does it differ from "diversity", etc? I would be grateful if the authors checked through their text to ensure that the reader can understand what is meant by these sometimes-loaded terms.

Answer: We agree that the term "variability" was not defined in the previous version of the article and using both "variability" and "diversity" is confusing. We had intended this term to encompass both specific MHC motifs and functional divergence. However since both are measures of diversity and as a consequence of this comment, we have decided to replace the term "variability" with "diversity" throughout the manuscript.

Page 3, lines 57-59. "... 29.4% of individuals tested AdV+ (n=143), 40.1% of 57 individuals (n=86) were infected with helminths, and 8.6% of individuals were co-infected with AdV and helminths...". 8.6% double infections is different from the product of the two single infections (11.8%), so I was curious whether this is a significant difference and whether it might be related some kind of exclusion (as mentioned for Campylobacter and adenovirus).

Answer: Many thanks for suggesting this and it is not something we considered before. In fact there is no statistical support for AdV infection to predict helminth infection (or *vice versa*) and this has been added in this new version of the article (lines 71-72).

Page 3, lines 59-63. "We detected a total of 226 MHCI functional alleles (median of 7 alleles per individual, range 4-20) in the population and grouped them into 17 MHC supertypes (ST) based on physicochemical variables (z-values [32]) of the amino acid sites affected by positive selection (PSS) (Materials and Methods, S1 Fig and S1 Table)." Having just complimented the authors on defining their terms, "functional allele" is another term with several meanings, but I see that I was pointed to the Materials and Methods, where the authors carefully describe what they mean. Still, it would be helpful to rearrange the sentence (if I have understood correctly) to reflect this: "We detected a total of 226 MHCI functional alleles (median of 7 alleles per individual, range 4-20) in the population based on physicochemical variables (z-values [32]) of the amino acid sites affected by positive selection (PSS) and grouped them into 17 MHC supertypes (ST) (Materials and Methods, S1 Fig and S1 Table)."

Answer: Indeed, the term was only previously defined in the Materials and methods and we have now edited the section suggested to define the term in the results as well (lines 72-76).

Perhaps a more important point is where are the sequences? In this age of supplementary materials, providing the original data on which the analyses are carried out is routine for biomedicine, and should be for this kind of (nearly) unique study. Perhaps the source of the sequences is mentioned somewhere, but I certainly didn't notice it.

Answer: We apologise for this omission, we had uploaded all sequences in NCBI prior to submission and this information was included in previous versions of the article but somehow got lost in the final version that we submitted. We have now included all sequences and NCBI accession numbers in the current version of this article under the section "Data and Code Availability".

As just one example of the benefits for the reader and the scientific community, I was unable to learn which sites are PSS in the S1 Table, since the basis of the numbering was not mentioned (is it from the start of the coding region including the signal sequence, it is from the presumed start of the mature protein; in both cases are they the same for all alleles detected; is the numbering based on the sequence of HLA-A2 as has been standard for decades or what exactly?).

Answer: We apologize for this omission. As suggested by Prof. Kaufman, we have now changed the numbering based on the HLA-A2 sequence and added this information in the legend of the Table S1.

Page 3, lines 63-65. "For the MHCII, we identified 49 functional alleles. A large number of MHCII alleles were rare (present in 1-2 individuals), therefore we used for subsequent analyses only alleles that were found in more than three individuals (S2 Fig)." Perhaps these alleles are rare for a reason (susceptibility?).

Answer: Perhaps but unfortunately as a consequence of the fact that these alleles are rare we do not have the power to test this hypothesis.

Page 3, line 69. Please define GLM (and other abbreviations) upon first usage. I was unable to discover where the term was defined in this paper, but I assume it stands for "generalised linear models".

Answer: Done.

Page 4, lines 95-96. "Individuals carrying the MHCII DRB allele Migr-DRB*34 are more likely to be AdVpos" is very correctly stated, since it is entirely possible that this class II allele is not causative for susceptibility, rather than a closely-linked gene with an entirely different function.

Answer: Thank you!

Page 4, lines 93-97. "We found a negative relationship between microbiome divergence and helminth infection status ... indicating that gut microbial communities across helminth infected individuals are more similar than the communities of non-infected individuals." Assuming that I have understood this correctly, the microbial communities are more similar between individuals infected with helminths compared to those not infected with helminths. So, one interpretation is that different pathogens drive the appearance of different microbial communities, so that "individuals not infected with helminths" could be infected with various other pathogens, each leading to a different microbial community and thus to the greater inter-individual differences in the microbial communities.

Answer: Yes, we completely agree and we have now added this point in the discussion (see lines 406-408). Many thanks for this insight.

Page 4, lines 116-118. "Our results revealed that sequence divergence at MHCIdistPSS ... (but not MHCInST) was a significant predictor of distance between samples in the constrained ordination space. " Perhaps I haven't understood the meanings of the terms correctly, but from my naïve perspective, different supertypes have different sequences in the peptide binding site, so having more supertypes would lead to more sequence divergence overall (that is, the two measures are correlated). If so, then this sentence points out an unexpected result that needs to be discussed further.

Answer: In our dataset the two measures are not correlated. We have now added a correlation plot to the supplementary material (Fig. S4) to illustrate the lack of collinearity between different measures of MHC diversity. To address this observation and as a result of a major comment from Prof. Kaufman, we added a section in Discussion concerning potential caveats of using number of supertypes as an informative estimate of functional diversity of the MHC (lines 326-335).

Page 4, lines 120-122. "We observed shifts in microbial composition according to the presence of eight MHCI supertypes (S5 Fig) and four MHCII alleles (S6 Fig)." Is there a reason not to show the microbial compositions of the supertypes and alleles for which the results were (apparently) not significant?

Answer: Yes, we chose not to do this because in total there are 17 MHCI supertypes and 20 MHCII alleles. Furthermore, the plots for non-significant supertypes/MHCII alleles are not very informative. Whilst we understand that the space in the supplementary material is unlimited, we feel that including figures for all (non-significant) supertypes and alleles would be excessive.

Page 5, lines 133-137. "We identified core taxa (prevalence $> 60\%$) associated with specific MHCI supertypes and MHCII alleles (using analysis of composition of microbes (ANCOM)) and that differed most with respect to their association with high or low estimates of MHC functional diversity (using a differential ranking approach) (see Materials and Methods)." Again my apologies for not understanding the terms used, but what is meant by "prevalence"? If a core taxon needs to be at least 60% of a microbial community, then there can't be more than one of them per individual.

Answer: We are unsure what is meant by this comment exactly. We think that there is a misunderstanding of the concept of prevalence across microbiome studies (see: Risely et. al. 2021, *Frontiers in Microbiology*). A prevalence value of 60% in this case means that a specific ASV is found in 60% of the individuals in the population or more (regardless of abundance). We have now clarified this in the text when we first mention prevalence thresholds of the core microbiome (Results section, lines 141-142).

Page 5, lines 138-140. "In fact, except for one MHCII allele (Migr-DRB*36) which was associated with seven core ASVs (and with 22 ASVs in total) . . . ". Please define abbreviations upon first use in text. I was unable to find a definition anywhere in the manuscript (and sadly I wasn't able to guess what it meant).

Answer: Done.

Page 5, lines 142-144. "This suggests that Migr-DRB*36 is a generalist [39] that indiscriminately binds a high number of antigens affecting both commensal and potentially pathogenic taxa." If I have understood this interpretation correctly, the number of taxa present in the microbial community of an individual would be considered to be due to direct recognition of antigens from those taxa. An alternative is that recognition of antigens from a taxon leads to exclusion (killing?); if this interpretation is correct, then DRB*36 would be a specialist binding antigens from only those taxa that are not present in such individuals.

Answer: Many thanks for this insight. Our understanding of the suggestion is that Migr-DRB*36 can be a specialist that may target a taxon with high influence on the microbial community and thus, by excluding it, it in turn shifts the relative abundance of other taxa. This is a good point and we have now added this alternative hypothesis to the main text (lines 152-158).

Page 5, lines 149-150. "We used Bifidobacteriums as a reference frame since this taxa was present in most samples . . . ". Isn't "taxon" the singular of "taxa"?

Answer: Indeed, thanks! We corrected this mistake.

Page 5, lines 151-160. "Our analyses indicate that resistance to AdV was provided by an increase in Campylobacter . . . ". I didn't see where the enrichment of Oridobacter in individuals with ST*2 is shown in Fig. 4A, or increase in Olsenella .... associated to MHCInST is shown in

Fig. 4C (and so on).

Answer: Here the incorrect figure was cited, it should have been figure 3 not 4. Many thanks for spotting this error.

Page 7, legend to Fig. 5. "Associations supported by model selection (AIC > 2) are reported with a red solid line, shaded area represent 95% confidence intervals." I found a red solid line only in C, so what are dashed black lines in A and B?

Answer: Dashed black lines represent non-significant trends. This is now mentioned in the legend.

Fig. 2. The y-axes of all the graphs in this figure are "infection probabilities", but the data is shown as one dot for each individual. It is difficult for me to understand how an individual has an "infection probability" since each individual was scored for being infected or not being infected. Please explain.

Answer: The individual data points (0 for uninfected and 1 for infected) are represented by the rug (as is standard in binomial models). Predicted values (i.e. the probably that that individual is infected according to the model) are represented by the dots. This is now explained in the legend of the figure to make this information clearer to the reader.

Figs. 3 and 5 (and S2, S3 and S5). Even with high resolution downloads and with zooming in, it was difficult for me to read some of the labels and designations. At least in the old days, there was a minimum font size for text in figures. Please increase font and choose colours so that the figure conveys the information as intended.

Answer: We apologize for this and following Prof. Kaufman's suggestions, we increased font size, modified the colour palette and created two Figures (Figure 5 and 6) that replaces Figure 5 which was overcrowded and difficult to read. We hope that the new version of the figures is now easier to read.

Reviewer #3

Part I – Summary

In this article Montero and colleagues investigate the links between diversity at MHC loci (class I and II), gut microbiome composition and infection by gut parasites (adenoviruses and helminths) in wild mouse lemurs. Importantly, the authors intended their paper to also address the question of potential indirect effects of MHC variation on parasite infection probability that would be mediated by the microbiome. By applying a broad range of statistical analyses they find that (i) a specific MHCII motif is associated with AdV positivity, (ii) MHCI variability as well as specific MHC I and II alleles influence microbiome composition, mostly by their effect on a few ASV abundance, (iii) among the ASV whose abundance was predicted by variation at MHC loci, some exhibited different relative abundance in individuals infected or not by gut parasites, (iv) most of these findings find a reasonable echo in analyses focused on bacterial functional pathways, not taxonomy.

This study addresses a consequential series of questions about the relationships between the immunogenome and the organisms it controls using a tri-partite data set (host, gut microbiome and gut parasites), which is still relatively rare. The paper reads well and I find the results very interesting, although, as acknowledged by the authors in the first part of the discussion, the nature of their study (observational and cross-sectional) prevents truly demonstrating indirect effects of MHC diversity on gut parasites mediated by the gut microbiome. In general, I'd be rather supportive of seeing this article published in PLoS Path. However, given its extensive use of a broad set of statistical tools I would find it reassuring to know it has been checked by a trained statistician (I am not this person). I also have a couple of major and minor remarks that the authors might address in a revision.

Answer: Many thanks for the very positive feedback and for the relevant suggestions from Dr. Sébastien Calvignac-Spencer.

Part II – Major Issues: Key Experiments Required for Acceptance

1) As afore mentioned the first part of the discussion is very careful about causality. In my view, it makes much sense to be careful since, in essence, their results are compatible with indirect effects mediated by the microbiome, but they are not a formal proof of the chain of causality. However, the authors still use a wording clearly suggestive of causality in multiple occasions throughout the ms. I did not compile a complete list but here a couple of examples, e.g. in the title, in the abstract "evidence of the indirect effect of the MHC via the microbiome" or l. 151.152 "resistance to AdV was provided by an increase in Campylobacter". It'd be great if the authors could carefully check through the ms that their wording is consistently careful.

Answer: We completely agree that interpreting causality should be treated with caution in a correlative study. We were trying to be very mindful of this in the first version but we agree that there were a few sections in the previous version of the manuscript that needed more careful wording. Addressing Prof. Jim Kaufman's comments (see above) has led us to be even more precise and careful with our interpretations of our results (for instance his comments regarding linkage of the MHCI and MHCII with other genes within the MHC region and our interpretation of MHCI supertypes). Dr. Calvignac-Spencer identifies specific sections that warrants careful wording, including the title, abstract, and author summary. We consider that the title is sufficiently cautious, since adding "Evidence" strongly nuisances causality. However, we agree that revision of other crucial sections of the main text were necessary and to address any further concern of over interpreting causality from correlations we changed the following sections:

- We changed the mentioned sentence in the abstract: "Among them were three taxa (*Odoribacter*, *Campylobacter* and Prevotellaceae-UCG-001) which were in turn linked to AdV and helminth infection status, correlative evidence of the indirect effect of the MHC via the microbiome."

- In the author summary we explicitly state that our lines of evidence are correlative. "Using a natural population of the primate *Microcebus griseorufus*, we provide correlative evidence of two outstanding findings: ..."
- The first sentence of the conclusion (lines 423-426.)

We have read through the manuscript carefully and we hope that in this version we are consistently careful regarding causality throughout.

2) The entire analysis relies on appropriately identifying MHC alleles. It is a complicated task with which the authors are very familiar. Here they identify many more MHCI than MHCII alleles. It turns out they also sequenced their MHCI amplicons much deeper than their MHCII. I imagine that this was maybe dictated by the structural differences they mention (with multiple paralogs of MHCI existing in mouse lemur genomes where MHCII is a single locus) and I also imagine that the workflow they designed and recently published can control for this, but it'd be good if the authors could show some data showing that these different depths could not explain the different allele numbers (e.g. sub-sampling their MHCI amplicons to the MHCII depth).

Answer: Many thanks for pointing out the potential pitfalls of appropriately genotyping the complex system that is the MHC and the importance of transparency. The target-specific primers that we used to amplify the MHCII-DRB gene do indeed amplify a single locus thus the maximum number of alleles for this gene is 2 (regardless of sequencing depth). On the other hand the primers we used for the MHCI amplified up to 10 loci and therefore the maximum number of alleles per individual is 20. It is common practice when genotyping a complex MHC system to increase sequencing depth to ensure that all alleles are captured during sequencing and allele calling (see Gillingham et al 2021 and Biedrzycka et al., 2017, both in *Molecular Ecology Resources*). Therefore, sequencing depth was substantially higher for MHCI than for MHCII. To formally evaluate this issue we tested whether allele calling per individual was predicted by sequencing depth. We did not find statistical support for this relationship either for the MHCI gene (GLMM, z=1.85, p-value = 0.065 when including an outlier in the model, and GLMM, z=1.36, p-value = 0.173 after removal of the outlier) nor the MHCII gene (GLMM, z=0.253, p-value = 0.800). This result is now reported in a new Figure in the Supplementary material (Figure S11). Furthermore, to address the Dr. Calvignac-Spencer's concerns we have now added in the methods (lines 529-532) that 85% of individuals were replicated for MHCII and 100% for MHCI, allele calling repeatability was 100% across both replicates. We hope that the addition of this information reassures the reader that there is no cause for concern regarding differences in sequencing depth, that the MHC genotyping methods used in our study are sound, and provides fuller transparency.

Part III – Minor Issues: Editorial and Data Presentation Modifications

l. 151-160: it is a bit unclear why the authors do not mention all groups whose Cohen's D 95% CI do not overlap with 0.

Answer: Given that our study is correlational and deals with indirect effects, we considered that it was very important to be conservative with our interpretations. Moreover, larger effect sizes are of greater biological and consequential interest than small effect sizes. Indeed, there is a tendency in the microbiome literature to over rely on significance (see Debelius et. al. 2016, *BMC genome biology*) at the cost of understanding (and reporting) the actual effect of a predictor. Therefore our selection criteria for further interpretation goes beyond whether Cohen's D 95% CI overlap 0 *per se* (since even small effects can be detected given a sufficiently large sample size) and we place emphasis on the size of the effect. This led us to focus on medium to large effect sizes (Cohen's D > |0.50|) to avoid interpreting potential false positives (type I errors after applying multiple testing corrections) and effects of little biological significance. We have now added a sentence to our methods to clearly state why we choose this strategy (lines 642-643).

l. 402: I assume but it is not stated clearly here that all the 143 anesthetized animals were

confirmed to be different individuals by genotyping?

Answer: Many thanks for spotting this omission. All animals were individually marked with electronic transponders and this ensured that each individual genotyped was unique. We have now added a sentence in the methods (line 453) stating this.
