# Supplementary material for: Evidence of MHC class I and II influencing viral and helminth infection via the microbiome in a non-human primate
Source: PLoS Pathog. 2021 Nov 8;17(11):e1009675. doi: 10.1371/journal.ppat.1009675 (PMC8601626; doi:10.1371/journal.ppat.1009675)
Supplement: Attachment — Submitted filename: SecondRebuttal_MHC_microbiome.pdf [file ppat.1009675.s021.pdf]

Universität Hamburg
DER FORSCHUNG | DER LEHRE | DER BILDUNG

B. Karina Montero, PhD

B. Karina Montero
Biozentrum Grindel
Martin-Luther-King Platz 3
Hamburg 20146, Germany
Phone: (+49)731 5022641
E-mail: b.karina.montero@gmail.com

September 26, 2021

Editorial board
PLOS Pathogens

Dear Editors,

On behalf of all co-authors I would like to once again thank you and the three reviewers for their positive feedback. We are very grateful for this transparent review process which greatly improved our manuscript. We have now addressed the outstanding concerns and would like to resubmit the revised version of our manuscript "**Evidence of MHC class I and II influencing viral and helminth infection via the microbiome in a non–human primate**" to PLOS Pathogens.

To address the concern from Reviewer 1 and following the suggestions by the editors, we have now added a new paragraph in the Discussion section which addresses in detail the limitations of using a natural primate population with regards to the potential confounding effects of previous infections on our results.

As in our previous response letter, our answers to raised concerns are marked in blue. We also provide a version of the manuscript that tracks the changes made to the text where changes are marked in blue.

Sincerely on behalf of all coauthors,

B. Karina Montero, PhD

Reviewer #1

Part I – Summary

The study focuses on an area of tremendous interest; the contribution of host genetic variability, particularly within the major histocompatibility complex, in shaping the composition of the gut microbial community. The premise is particularly important and the authors combine modern laboratory techniques with field work, which is both novel and highly interesting.

Answer: We would like to thank again the reviewer for their positive comments. We hope that we have now addressed the concern raised regarding the potential influence of previous infection on immunity.

Part II – Major Issues: Key Experiments Required for Acceptance

The authors have not addressed the possible confounding effect of pre-existing immunity (due to prior non-persistent exposure) to AdV or helminths. They simply removed mention of viral persistence from the manuscript to get around my comment. The argument being made in the manuscript is that at this specific moment in time, these specific animals are infected/colonized with these specific "parasites", because of supposed crosstalk between host MHC, the gut flora, and these "parasites".

The authors discuss everything (e.g., T-regs, complement, thymic selection, TCR) except the confounding effect of prior exposure on the stratification of the study population into groups.

There must be three or four biologically distinct groups:

- A infected; no prior exposure
- B. infected; prior exposure
- C. uninfected; no prior exposure
- D. uninfected; prior exposure This manuscript only assumes A and C.

To make claims about modified susceptibility to infection and not at all discuss or show data speaking to prior exposure and pre-existing immunity seems disingenuous at worst and a major oversight at best.

At minimum, add an entire paragraph to the discussion that incorporates how prior exposure and pre-existing immunity to AdV and helminths might confound your interpretation.

Answer: Unfortunately, our study design does not allow to infer previous infection history due to our non-invasive sampling strategy of a primate species (infection status was determined from fecal samples and no blood samples were taken). We understand the need to discuss the influence of previous infections on current infection status and its potential confounding effects on our results. We now do so with a new last paragraph of our discussion (lines 422-441).

Reviewer #2

Part III – Minor Issues: Editorial and Data Presentation Modifications

Many thanks to the authors for considering my comments and for the modifications which I believe strengthened an already fine paper. I have only two more comments.

Answer: We would like to thank Prof. Kaufmann once again for his positive feedback.

Lines 163-164 of the revised manuscript: Either "this taxon was" or "these taxa were", please.

Answer: Done!

Lines 660-661 of the revised manuscript: many thanks for the kind acknowledgement, but I think that is only one anonymous reviewer.

Answer: Indeed, many thanks for spotting this error!

Reviewer #3

Part I – Summary

The authors have appropriately addressed all my concerns. It is a nice piece that in my view warrants publication in PLoS Pathogens.

Answer: Once again, we are very grateful to Dr. Sébastien Calvignac-Spencer for his positive feedback.
